# β–Lactam TRPM8 Antagonist RGM8-51 Displays Antinociceptive Activity in Different Animal Models

**DOI:** 10.3390/ijms23052692

**Published:** 2022-02-28

**Authors:** Cristina Martín-Escura, Alicia Medina-Peris, Luke A. Spear, Roberto de la Torre Martínez, Luis A. Olivos-Oré, María Victoria Barahona, Sara González-Rodríguez, Gregorio Fernández-Ballester, Asia Fernández-Carvajal, Antonio R. Artalejo, Antonio Ferrer-Montiel, Rosario González-Muñiz

**Affiliations:** 1 Instituto de Química Médica (IQM-CSIC), 28006 Madrid, Spain; cristinamartinescura@gmail.com (C.M.-E.); lukeanthonyspear@gmail.com (L.A.S.); 2 Alodia Farmacéutica SL, 28108 Alcobendas, Spain; 3 IDiBE, Universidad Miguel Hernández, 03202 Elche, Spain; mepea8@gmail.com (A.M.-P.); rober.dltm@gmail.com (R.d.l.T.M.); saragonzalezrodriguez@gmail.com (S.G.-R.); gregorio@umh.es (G.F.-B.); aferrer@umh.es (A.F.-M.); 4 Departamento de Farmacología y Toxicología, Facultad de Veterinaria, Universidad Complutense de Madrid, 28040 Madrid, Spain; olivos@ucm.es (L.A.O.-O.); vbg@ucm.es (M.V.B.); antonio.artalejo@vet.ucm.es (A.R.A.); 5 Instituto Universitario de Investigación en Neuroquímica, Universidad Complutense de Madrid, 28040 Madrid, Spain

**Keywords:** TRPM8 channels, antagonist, β–lactam, oxaliplatin-induced peripheral neuropathy, CCI chronic neuropathic, nociception, NTG-induced hyperesthesia

## Abstract

Transient receptor potential melastatin subtype 8 (TRPM8) is a cation channel extensively expressed in sensory neurons and implicated in different painful states. However, the effectiveness of TRPM8 modulators for pain relief is still a matter of discussion, since structurally diverse modulators lead to different results, depending on the animal pain model. In this work, we described the antinociceptive activity of a β–lactam derivative, RGM8-51, showing good TRPM8 antagonist activity, and selectivity against related thermoTRP channels and other pain-mediating receptors. In primary cultures of rat dorsal root ganglion (DRG) neurons, RGM8-51 potently reduced menthol-evoked neuronal firing without affecting the major ion conductances responsible for action potential generation. This compound has in vivo antinociceptive activity in response to cold, in a mouse model of oxaliplatin-induced peripheral neuropathy. In addition, it reduces cold, mechanical and heat hypersensitivity in a rat model of neuropathic pain arising after chronic constriction of the sciatic nerve. Furthermore, RGM8-51 exhibits mechanical hypersensitivity-relieving activity, in a mouse model of NTG-induced hyperesthesia. Taken together, these preclinical results substantiate that this TRPM8 antagonist is a promising pharmacological tool to study TRPM8-related diseases.

## 1. Introduction

The cold-temperature thermosensor TRPM8 is a non-selective cation channel, widely expressed in peripheral sensory neurons having their soma in the dorsal root (DRG) and trigeminal (TG) ganglia, but also in different organs and tumor malignancies [1]. The important role of TRPM8 channels in the pathophysiology of diverse biological processes fosters the development of agonist/antagonist modulators, to enlarge the arsenal of pharmacological tools to study TRPM8-related diseases, including inflammatory and neuropathic disorders, as well as tumor growth and dissemination [1]. In fact, a number of agonists and antagonists, belonging to different chemical families, have already been described [2,3].

The high expression of TRPM8 channels both in sensory neurons and organs, in addition to their activation by cold, point to these channels as mediators of cold-induced nociception and cold-hypersensitivity developed in certain peripheral neuropathies [4]. Nevertheless, some discrepancies have been found in the literature, and the exact role of TRPM8 in different pain types, and the usefulness of TRPM8 modulators to relieve them, is still a matter of profuse research.

The chronic constriction injury of the sciatic nerve (CCI) is a model of neuropathic pain that courses with hypersensitivity to cold, heat and mechanical stimuli [5]. Initial investigations on the expression of TRPM8 and TRPA1 channels after CCI injury, identified a low expression level of both channels in DRG neurons, and discard their involvement in cold hypersensitivity [6]. However, other experimental evidence indicates that sensitivity to cold after CCI is aggravated by TRPM8 activation and the increased expression of these channels in DRG neurons, whereas their downregulation or pharmacological blockade ameliorates CCI-induced cold hypersensitivity [7,8,9]. In good agreement, it has been described that the intrathecal administration of the TRPM8-selective antagonist AMTB reduces cold hypersensitivity in rats, but it exacerbates thermal nociception and has no apparent influence on mechanical hypersensitivity in the CCI model [10]. Likewise, the TRPM8 antagonist AMG2850, displaying a potent nanomolar profile in vitro, was able to reduce the number of wet-dog-like shakes induced by icilin, a TRPM8 agonist, but was ineffective on mechanical hypersensitivity in the complete Freund’s adjuvant (CFA) model of inflammatory pain and the spinal nerve ligation model of neuropathic pain [11]. The authors suggest that TRPM8 channels could not have a relevant role in nociception in this model, but this is in contrast with recent results found for the novel TRPM antagonists DFL23693 and DFL23448 (imidazo[1′,5′:1,6]pyrido[3,4-*b*]indole-1,3(2H)-dione derivatives), which indicate that they effectively relieve thermal and mechanical hypersensitivity in CCI rats [12,13].

Thermal and mechanical hypersensitivity are also prominent symptoms in the peripheral neuropathy developed after chemotherapeutic treatments for cancer (i.e., platinum-derived drugs, vinca alkaloids and taxanes) [14] or viral infections (HIV, HCV, HSV, HZV) [15,16]. Some channels of the TRP family expressed in nociceptors, like TRPV1, TRPV4, TRPA1 and TRPM8 seem to contribute to this painful, exacerbated sensitivity [17]. For instance, oxaliplatin (OXA) causes acute and chronic peripheral pain, characterized by cold hypersensitivity, and seems to be related to increased activity of the TRPM8 channels, linked to the activation of phospholipase C and reduction in phosphatidylinositol 4,5-bisphosphate production [18]. In primary sensory neurons, the overexpression of TRPM8 mRNA and protein in oxaliplatin-induced peripheral neuropathy seems also to be mediated through the c-Myc regulatory gene [19]. Moreover, in both short- and long-term oxaliplatin-induced neuropathy animal models, both TRPA1 (HC033031) and TRPM8 (AMTB) antagonists attenuated the initially observed vasodilation after cold exposure [20]. Similarly, a recent study describes that oxaliplatin causes excitability of IB4 neurons, which can be attenuated by antagonists of TRPA1 (A-967079) and TRPM8 (TC-I), but not by capsazepine, a TRPV1 blocker, suggesting the contribution of cold channels in neuronal sensitization [21]. However, only the pretreatment with TC-I prevented cold and mechanical hypersensitivity induced by oxaliplatin, denoting key importance of the TRPM8 channel in the initial phase of sensitization. The involvement of L-type voltage-gated Ca^2+^ channels in the over-expression of TRPM8 receptors leading to oxaliplatin-associated peripheral neuropathy has also been reported [22].

Cold hypersensitivity is also referred to as an important, undesirable secondary effect by patients receiving chronic treatment with opioids [23]. The hypersensitivity after sustained morphine administration was linked to TRPM8 upregulation, and can be blocked by TRPM8 antagonists, while TRPM8 knockout mice are not able to develop it [24].

Migraine, a neurovascular disorder affecting more than 14% of the adult population and more frequent in women than in men, is one of the most disabling conditions in the world. Some experimental evidence indicated that TRPM8 channels are overexpressed in a rat model of migraine, and that these channels could be implicated in the migraine mechanism, possibly through activation of NMDA receptors [25]. In addition, among other genetic factors, TRPM8 single-nucleotide polymorphisms (SNPs), such as rs10166942 and rs11562975, have been associated with chronic migraine and connected to allodynic symptoms [26,27,28]. Similarly, the SNP rs2651899 was described as a possible genetic marker for migraine susceptibility [27]. Concerning described TRPM8 antagonists, AMG 333 reached phase 1 clinical trials for the treatment of migraines [29]. However, the important side effects reported by volunteers, consisting of paresthesia, dysesthesia, dysgeusia and intolerable hot feeling, led to the compound’s withdrawal, preventing its further progress to later clinical studies.

**Figure 1:**
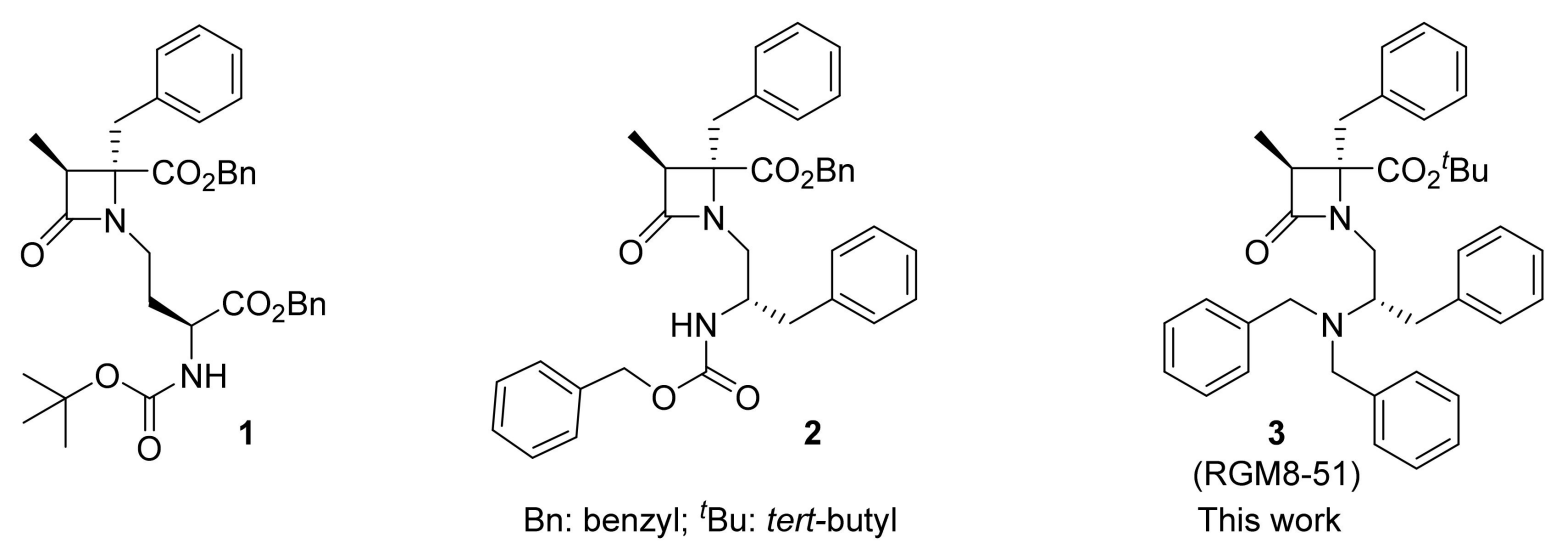
β–lactam TRPM8 antagonists: Compounds **1** and **2** came from previous works [30,31]; Compound **3** (RGM8-51), described in this work.

A few years ago, we described a new series of β–lactams acting as potent and selective TRPM8 antagonists (i.e., compounds **1**, **2**, Figure 1) [30,31], with compound **2** displaying significant activity for relieving oxaliplatin-induced cold hypersensitivity [31]. To advance in the characterization of our β–lactam derivatives, we selected a prototype in the series, RGM8-51 (**3**) for parallel evaluation in different animal models of pain occurring with upregulation of TRPM8 channels. Here, we describe the in vitro properties of RGM8-51 as a blocker of the TRPM8 activation and its selectivity over other TRP channels and receptors related to pain. In addition, we report the results of its in vivo activity in a mouse model of oxaliplatin-induced peripheral neuropathy, in the CCI model of neuropathic pain, and in NTG-induced hyperesthesia.

## 2. Results and Discussion

### 2.1. Synthesis

**Scheme 1:**
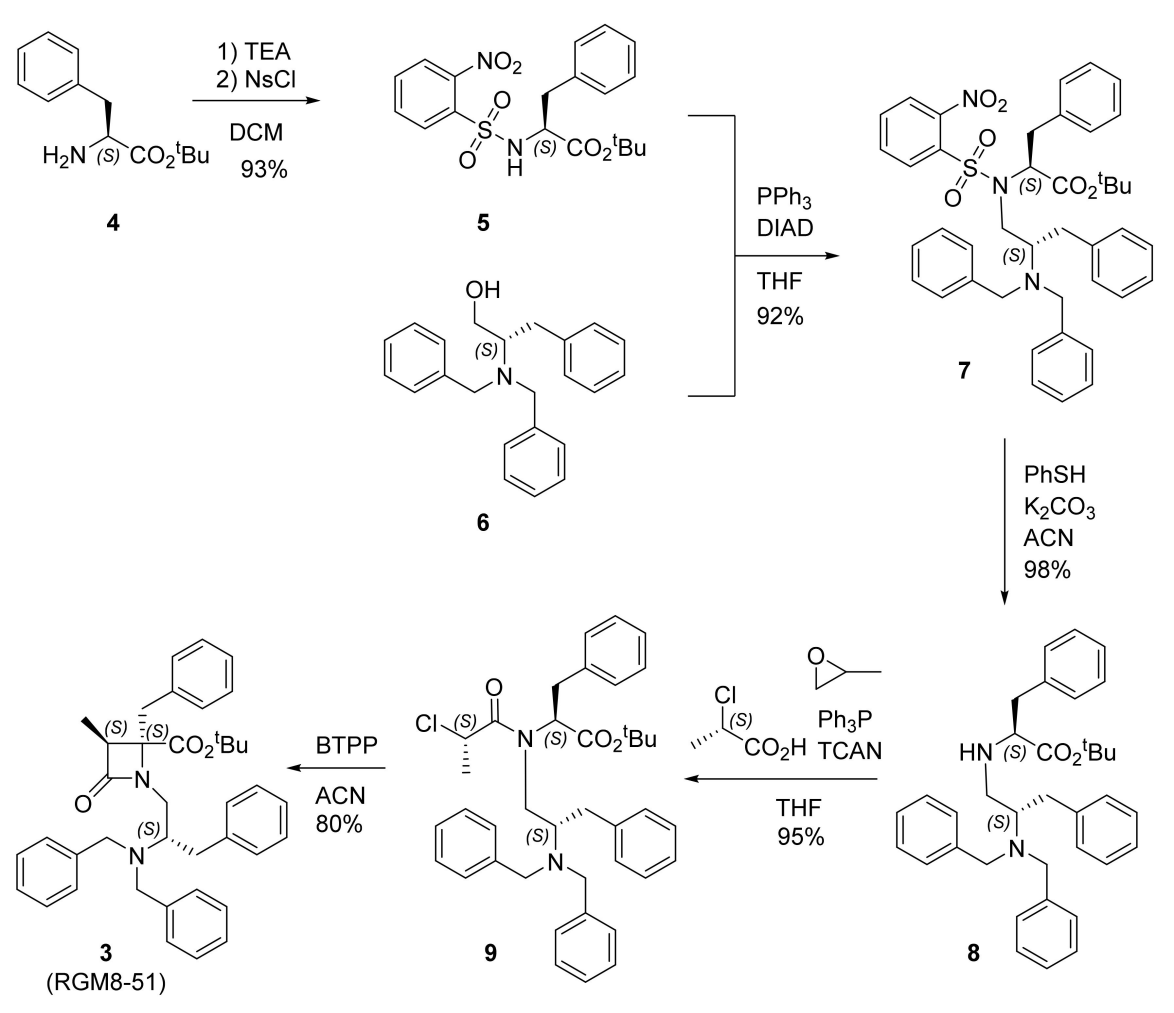
Synthetic procedure for the preparation of β–lactam **3** (RGM8-51) form H-L-Phe-O*^t^*Bu (**4**). Compounds **5**–**9** are the synthetic intermediates in the process.

The synthetic multistep procedure to prepare β–lactam **3** (RGM8-51) is depicted in Scheme 1. Briefly, the commercial amino acid derivative HCl·H-L-Phe-O*^t^*Bu (**4**) is reacted with nosyl chloride to afford *N*-Ns-L-Phe-O*^t^*Bu (**5**) in very good yield. Under Mitsunobu conditions, using triphenylphosphine and diisopropylazido dicarboxylate (DIAD), amino acid derivative **5** reacts with 2*S*-(dibenzylamino-3-phenyl)propanol (**6**) to give the Phe-phenylalaninol conjugate **7** in excellent yield. Subsequently, the removal of the nosyl group, carried out with PhSH in a basic medium, led to the secondary amine **8**, almost quantitatively. Then, an acylation reaction with 2*S*-chloropropanoyl chloride, prepared in situ from 2*S*-chloropropanoic acid, trichloroacetonitrile and triphenyl phosphine, allowed the formation of the enantiopure chloropropanoyl derivative **9**. Finally, the BTPP-assisted cyclization of intermediate **9** gave rise to the desired β–lactam **3**, with an overall yield of 64%. A 3*S*,4*S*,2′*S* configuration was assigned to compound **3**, since the configuration of the 2-chloropropanoic acid used, 2*S*, directs the exclusive formation of the 3*S*,4*S* β–lactam ring, as previously demonstrated [32].

### 2.2. In Vitro TRPM8 Antagonist Activity

#### 2.2.1. Experiments in Cell Lines

Calcium (Ca^2+^) micro-fluorography assays were performed on HEK293 cells that heterologously express both rat (*r*) or human (*h*) TRPM8 channel isoforms, using menthol as activation stimulus [30]. β–lactam RGM8-51 (**3**) did not have any ability to activate TRPM8 channels, but produced a concentration-dependent inhibition of menthol activation. As indicated in Table 1, compound RGM8-51 displays antagonist activity at both isoforms of the TRPM8 channel. The IC_50_ values are in the micromolar range, 1.06 ± 1.21 and 1.74 ± 1.19 μM, respectively, slightly higher for *h*TRPM8. This represents a similar activity as β–lactam **2** and a somewhat higher potency than the model TRPM8 antagonists AMTB.

**Figure 2:**
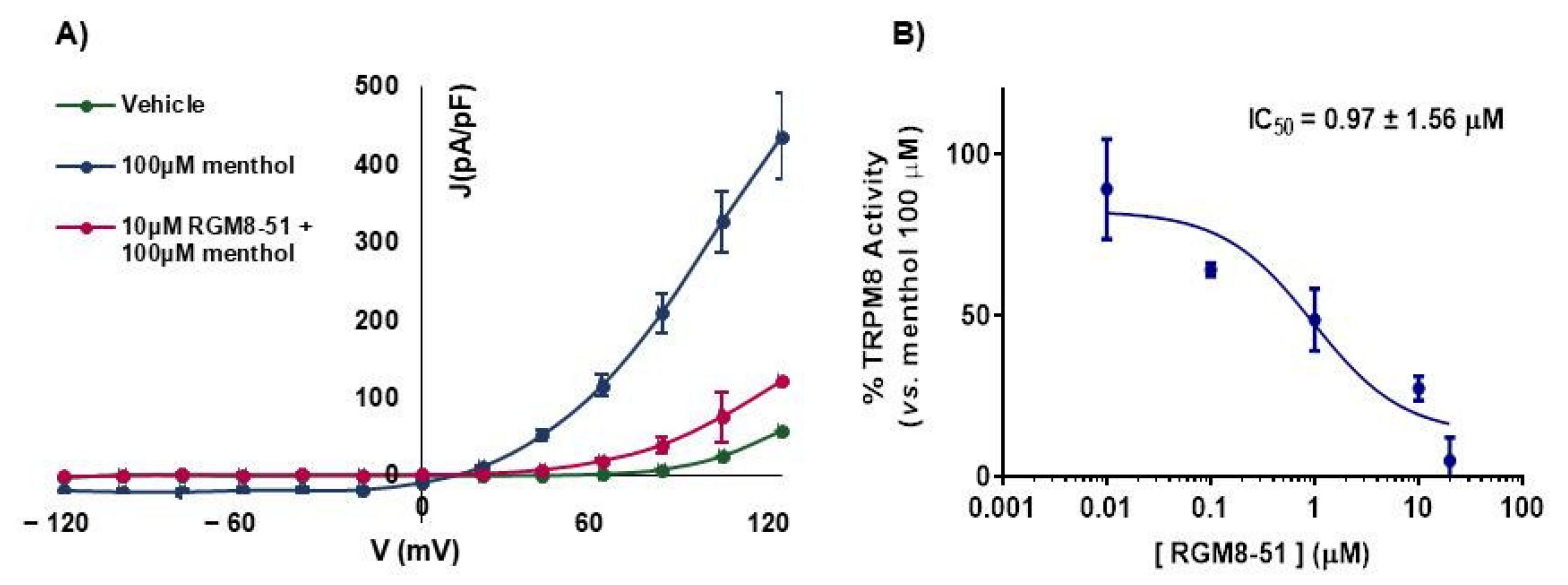
Compound **3** (RGM8-51) blocks TRPM8-mediated responses evoked by menthol in *r*TRPM8-expressing HEK293 cells. (**A**) IV curves obtained after exposure to vehicle solution (green trace), 100 μM menthol (blue trace), and 100 μM menthol + 10 μM RGM8-51 (red trace). Peak current data were expressed as pA/pF (to allow comparison among different size cells). Each point is the mean ± SEM of n = 15. (**B**) Concentration/response curves for *r*TRPM8 current blockade by RGM8-51, at a holding voltage of −60 mV. The solid line represents fits of the experimental data to the following binding isotherm: y = max/(1 + x/IC_50_)n, where x is the drug concentration and n the Hill coefficient. The fitted value for IC_50_ was 0.97 ± 1.56. Each point is the mean ± SEM of n = 15.

To confirm the antagonistic activity, electrophysiology (patch-clamp) studies were performed on HEK293 cells expressing the *r*TRPM8 channel. As shown in Figure 2, perfusion with 100 µM menthol (blue line) produces a strong outward rectifying ionic current, characterized by a negligible current at negative potential and a linear increase (ohmic behavior) at positive voltages. This current is clearly reduced by addition of β–lactam RGM8-51 (10 μM, red line). Then, using a holding voltage of −60 mV, different concentrations of RGM8-51 were used to establish the concentration/response curve for *r*TRPM8 current blockade. The obtained IC_50_ value, 0.97 ± 1.56 μM (Table 1), confirms a micromolar antagonist potency.

To uncover the selectivity of RGM8-51 for the TRPM8 channel, its activity was measured on different ion channels and receptors involved in pain-related processes. Firstly, *h*TRPV1, *h*TRPV3 and *h*TRPA1 ion channels were selected, since they are expressed in primary afferent neurons, and involved in the integration of temperature and nociception [34,35]. The *h*ASIC3 channel was also included in the selectivity study because it is widely distributed in the peripheral nervous system, and its activation augments excitability of primary sensory neurons [36].

In all cases, the compound was evaluated at 10 µM, a concentration that is ~10 and >5 times higher than the IC_50_ value for *r*TRPM8 and *h*TRPM8, respectively. As indicated in Table 2, compound RGM8-51 shows some ability to activate the *h*TRPV1 channel (~30%), while its activity as an antagonist at this heat receptor is lower than 20%. β–lactam RGM8-51 did not display any agonist or antagonist profile in cool-activated TRPA1 channels and has negligible activity as TRPV3 and ASIC3 channel antagonists. In general, this β–lactam is selective for cold-activated TRPM8 channels, although TRPV1 channels could be activated to some extent at high concentrations.

Next, the assessment of activity at other peripheral receptors related to pain, inflammation or migraine was considered. Calcitonin gene related peptide (CGRP) receptors are expressed in numerous tissues and in primary sensory neurons of TGG, where they are involved in pain transmission and inflammatory processes [37]. CGRP levels have also been shown to increase in cranial circulation of patients with migraine symptoms [38]. Cannabinoid receptor CB2 is highly expressed in the peripheral nervous system, where it is rapidly regulated in peripheral neurons after injury and inflammation to dampen neuronal excitability and has been associated with paclitaxel-induced peripheral neuropathy [39,40,41]. Muscarinic acetylcholine receptors (mAChRs) are mainly located at the dorsal horn of the spinal cord and also seem to contribute to nociceptive regulation [42]. Among mAChRs, the M3 subtype is also expressed in different types of skin cells, such as keratinocytes and melanocytes [43,44].

To define whether β–lactam derivative RGM8-51 could bind to CGRPR, CB2 and M3 receptors, in vitro binding assays were performed, using CHO cell lines transfected with these receptors. The degree of binding was determined by using specific radioligands for each receptor (Table 2, footnote). As shown in Table 2, RGM8-51 at a 10 μM concentration did not show significant affinity for the mentioned receptors, thus reinforcing its selectivity for TRPM8 channels.

#### 2.2.2. Experiments in DRG Neurons

**Figure 3:**
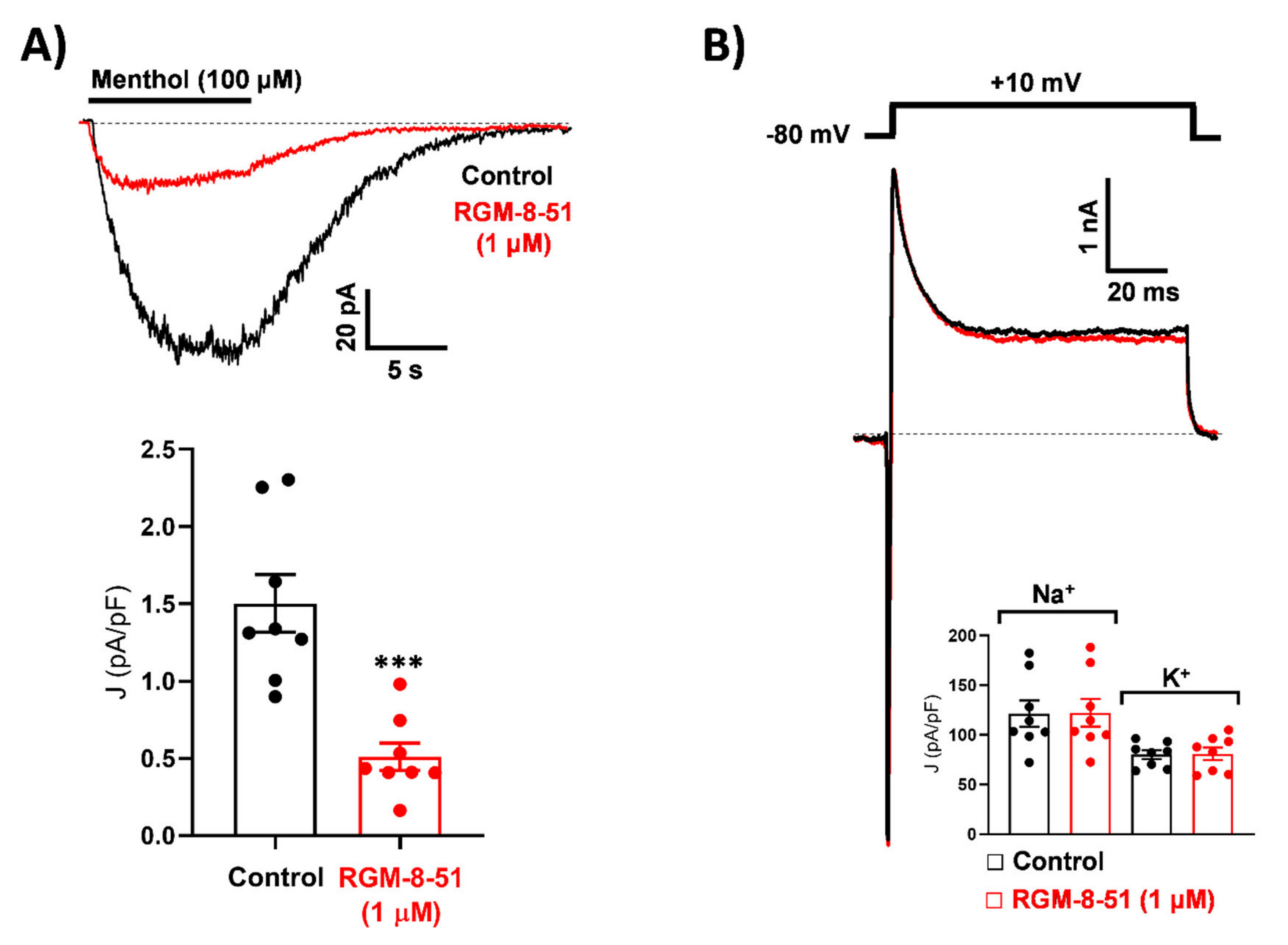
Effect of RGM8-51 on TRPM8-mediated and voltage-activated Na^+^ and K^+^ currents in rat DRG neurons. Currents were evoked by menthol (100 µM) (**A**) or a voltage depolarization to +10 mV (**B**). The horizontal bar on top of the recordings in A indicates the time period of menthol application. Holding voltage was −80 mV. Bar/scatter plot graphs in the lower part of the panels show peak-current densities (J; pA/pF) before and after RGM8-51 (1 µM). Data are mean ± S.E.M (n = 8 cells from 5 cultures). Statistical analysis was performed by using the paired *t*-test. *** < 0.001.

We also investigated the effect of RGM8-51 on native TRPM8 channels from rat DRG neurons. Whole-cell patch-clamp recordings from small size DRG neurons evidenced menthol (100 µM)-activated inward currents at a holding voltage of −80 mV (Figure 3A). As expected from results obtained in *r*TRPM8-expressing HEK293 cells, RGM8-51 (1 µM) inhibited menthol-activated currents. Mean peak current inhibition amounted to 65.01 ± 4.67% (from 1.50 ± 0.19 to 0.51 ± 0.09 pA/pF; n = 8 cells) (Figure 3A). Interestingly, RGM8-51 did not modify on its own the baseline current at -80 mV and had no effect on voltage-activated Na^+^ and K^+^ currents evoked by a depolarization pulse to +10 mV (Figure 3B). Besides extending results obtained with RGM8-51 in heterologously expressed TRPM8 channels to native TRPM8 channels, these data rule out a direct effect of this compound on major ion conductances responsible for action potential generation in primary nociceptive neurons.

**Figure 4:**
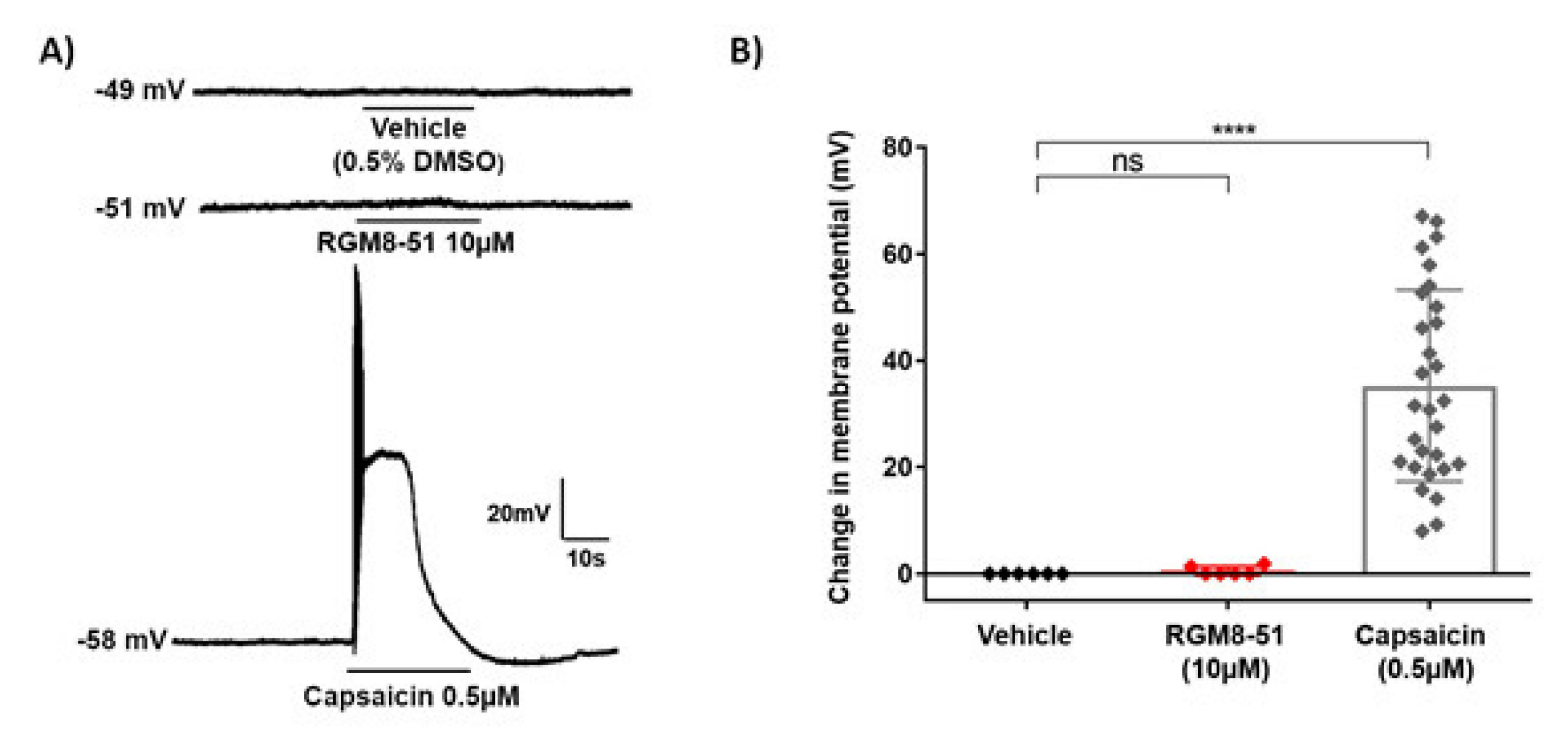
Compound RGM8-51 does not affect the membrane resting potential of sensory neurons. (**A**) Representative recordings of resting membrane potential measured under current-clamp conditions. The upper trace shows how the resting membrane potential remains constant after the addition of vehicle, and 10 μM of compound RGM8-51 (middle trace). The bottom trace shows action potentials firing in a DRG neuron exposed to 0.5 μM capsaicin. (**B**) Changes in membrane potential in the absence and the presence of 10 μM of compound RGM8-51, and in the presence of capsaicin (Cap) 0.5 μM. Data were analyzed by one-way ANOVA followed by Bonferroni post hoc test for multiple comparisons (ns = no significance, **** = 0.0001) and given as mean ± SEM; n ≥ 6 cells from 2 cultures.

In order to test if compound RGM8-51 could affect the membrane resting potential (MRP) of DRG sensory neurons, we also measured MRP of sensory neurons in the current-clamp mode of patch-clamp. In this condition, small sensory neurons displayed a MRP of ~−50 mV (Figure 4A, top trace, and Figure 4B). Exposure of the neurons to 10 μM of RGM8-51 did not alter the MRP of these neurons (Figure 4A, middle trace, and Figure 4B). As control to ensure that neurons were able to exhibit action potential firing they were exposed to 0.5 μM capsaicin (Figure 4A, bottom trace, and Figure 4B).

#### 2.2.3. In Vitro Preliminary Pharmacokinetic Studies

RGM8-51 displayed quite good solubility (198 μM) in simulated gastric conditions (pH < 5.5). This compound has a logD of 5.7 and was not able to permeate CACO-2 cell monolayers, indicating that oral administration cannot be selected for in vivo experiments (Table S1). RGM8-51 shows high binding to serum proteins (99–100%). As expected by the high number of phenyl rings, the ester moiety and the N-benzyl groups, neither the stability in human hepatic microsome cultures is high (t_1/2_ = 11 min) nor the intrinsic clearance is very good (Clint = 614 μL/min/mg), although the value is better than that of terfenadine, used as one of the reference controls. As indicated later, despite not optimal PK properties, RGM8-51 displayed significant activity in in vivo experiments after intraplantar (i.pl.) and intraperitoneal administration (i.p.).

### 2.3. Molecular Modeling Studies

The identification of putative binding pockets for RGM8-51 within the TRPM8 tetrameric channel was performed by docking studies simulations, using Yasara software [45,46], in a 3D model of the *r*TRPM8 channel. This model was constructed from the cryo-electron microscopy (cryo-EM) structure of the bird *Ficedula albicollis* TRPM8 (*fa*TRPM8, PDB code 6BPQ) [47].

**Figure 5:**
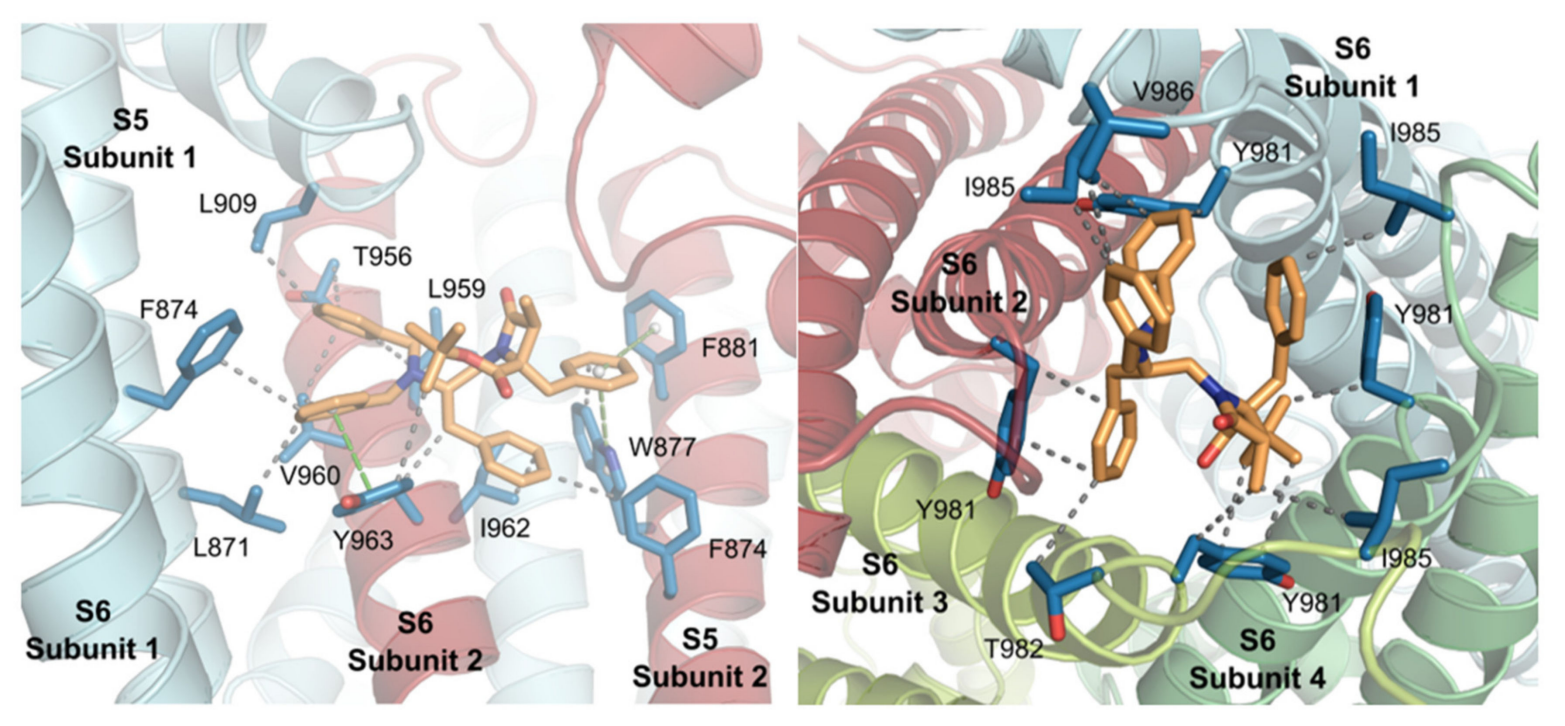
Interaction of β–lactam RGM8-51 (carbon atoms in wheat color) at subsite 1 (**left**) and subsite 2 (**right**). Channel residues involved in the interactions are shown in blue. WdV interactions are indicated by doted gray lines, and π–π stacking by centroids and non-continuous pale green lines.

Four main solutions were found for the accommodation of RGM8-51 in the TRPM8 channel, most of them located by the pore zone (Table 3). The most populated subsite 1 is a cavity formed by residues at S5-S6 transmembrane helices, located at the upper part of a protein subunit, and the S5 and the S5-to-S6 pore loop of a contiguous subunit (Figure 5, left). As for the concrete interconnections, three incidences of π−π stacking were identified. Two of them involve the aromatic ring of the 4-Bn group of RGM8-51 and Trp877 and Phe881 of the channel, and are T-shape interactions, while the third one is a face-to-face π-π stacking between the small molecule N-Bn group and the Phe 963 of the protein. In addition, a high number of Van der Waals interactions contribute to the stabilization of the complex, including the *tert*-butyl group on the β–actam and Tyr963, the CH_2_ of the 4-Bn and the CH_2_ of Tyr963, the 2′-Bn group and Phe874 and Ile963, and the N-Bn_2_ and Leu871, Phe 874, Leu909, Thr956 and Val960.

The second subsite locates the small molecule at the very bottom part of the pore, interacting with the loops joining S6 and TRP domain of the four channel subunits (Figure 5, right). In this case, the interplay is mediated by a network of Van der Waals (VdW) hydrophobic interactions, involving all substituents on the β–lactam. Thus, the *tert*-butyl group interacts with both the CH_2_ of Tyr981 in subunit 1 and the aromatic ring of Tyr981 in subunit 2. This latter Tyr residue also interacts with the 3-Me group, as does Leu985 of the same subunit. The 2′-Bn moiety connects to Thr982 in protein monomer 2 and Tyr981 of subunit 3. Finally, the tertiary N-Bn_2_ moiety establishes contacts with Tyr981, Ile985 and Val986, all in monomer 4.

In addition, there are two other possible binding sites. The third subsite where the molecule interacts with the channel is a cavity formed by S3 and S4 from a subunit and S6 from the adjacent subunit, at the top part of this one. The fourth pocket is located between the two small alpha helices that join S5 and S6 of the same subunit, at the top of the ion channel. Although they are also possible binding sites, they were less populated in the modeling studies and yielded lower binding energies than subsites 1 and 2 (Table 3).

The structure of TRPM8/AMTB and TRPM8/TC-I2014 complexes reveals interconnection of these small molecules by the menthol binding site, revealed as a highly adaptable pocket [48]. Our modeling studies, once again, point to a different mode of interaction of our β–lactam derivatives with respect to other described TRPM8 antagonists, making them a unique class within described modulators.

### 2.4. In Vivo Antinociceptive Activity

RGM8-51 was further used as a chemical tool to explore the role of TRPM8 in different animal models of pain in vivo, including CIPN, CCI model of neuropathic pain, and NTG-induced mechanical hypersensitivity.

#### 2.4.1. Mouse Chemotherapy-Induced Peripheral Neuropathy

It is known that the painful peripheral neuropathy associated with the use of the chemotherapeutic drug oxaliplatin (OXA) is intensified by cold [49], and that TRPM8 channels play an important role in this debilitating condition [18,21]. In fact, the OXA-induced peripheral neuropathy mice model is being increasingly used to characterize TRPM8 antagonists. For instance, an imidazo[1′,5′:1,6]pyrido[3,4-*b*]indole-1,3(2*H*)-dione derivative, acting as a potent nanomolar TRPM8 antagonist, inhibited OXA-induced cold hypersensitivity after intraplantar (i.pl.) administration of 10 and 30 μg of compound, with remarkable effect after 15 min and up to 30 min [12]. Of special interest is a biphenylamide *N*-spiro[4.5]decan-8-yl derivative that showed nanomolar potency on human TRPM8, and attenuated cold allodynia from 15-60 min at 0.1 μg (i.pl.) dose, maintaining a significant effect up to 60 min after administration of a 1μg dose [50].

**Figure 6:**
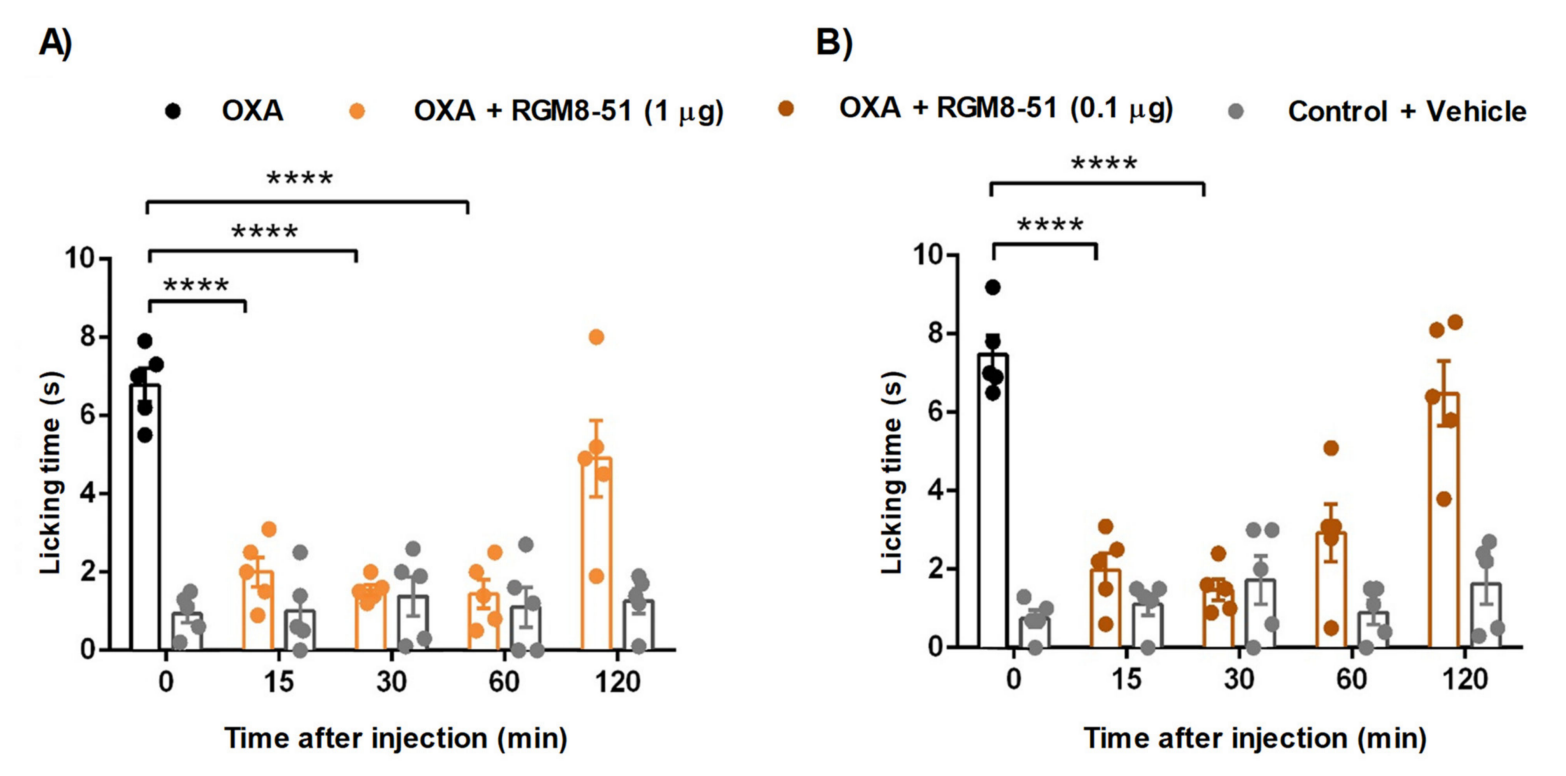
Effects of compound RGM8-51 on the oxaliplatin (OXA)-induced cold hypersensitivity (drop acetone test). Peripheral neuropathy was induced in male mice by injecting OXA (6 mg/kg, s.c.) on days 1, 3 and 5. Control animals were treated with vehicle. Compound RGM8-51 (1 µg/i.pl., (**A**) or 0.1 µg/i.pl., (**B**)) was administered to the OXA-treated animals, and the time-course of cold hypersensitivity was measured. Data are given ± SEM (n = 5). Statistical analysis, comparing with oxaliplatin-treated mice, was performed by two-way ANOVA followed by post hoc Bonferroni test by multiple comparisons: **** *p* < 0.0001.

For the OXA-induced peripheral neuropathy assay, OXA was subcutaneously (s.c.) injected on days 1, 3 and 5 to male mice, at a 6 mg/kg dose, to induce hypersensitivity to cold, as monitored by the acetone drop test (Figure 6).

Compound RGM8-51 was administered intraplantarly (i.pl., 1 and 0.1 μg), to evaluate its potential to reduce the OXA-induced cold hypersensitivity. As shown in Figure 6A, RGM8-51 (1 μg) attenuates the cold-induced paw licking in a significant manner 15 min after administration, showing the peak effect from 30 to 60 min. The compound is still highly active at a 0.1 µg dose, again with a sustained activity from 15 to 30 min. Compared to compound **2** [31], β–lactam analogue RGM8-51 needs lower doses (more potent) to reduce cold hypersensitivity in a significant manner. The potency is also higher than that described for imidazo[1′,5′:1,6]pyrido[3,4-*b*]indole-1,3(2H)-dione derivatives [12], and similar to the best biphenylamide-spiro derivative [50]. The period of time in which RGM8-51 effectively decreases cold allodynia is higher than that described for an imidazo[1′,5′:1,6]pyrido[3,4-*b*]indole-1,3(2H)-dione heterotricyclic TRPM8 antagonist [12], despite the in vitro potency being lower in our case.

#### 2.4.2. Chronic Constriction Injury (CCI) of the Sciatic Nerve

**Figure 7:**
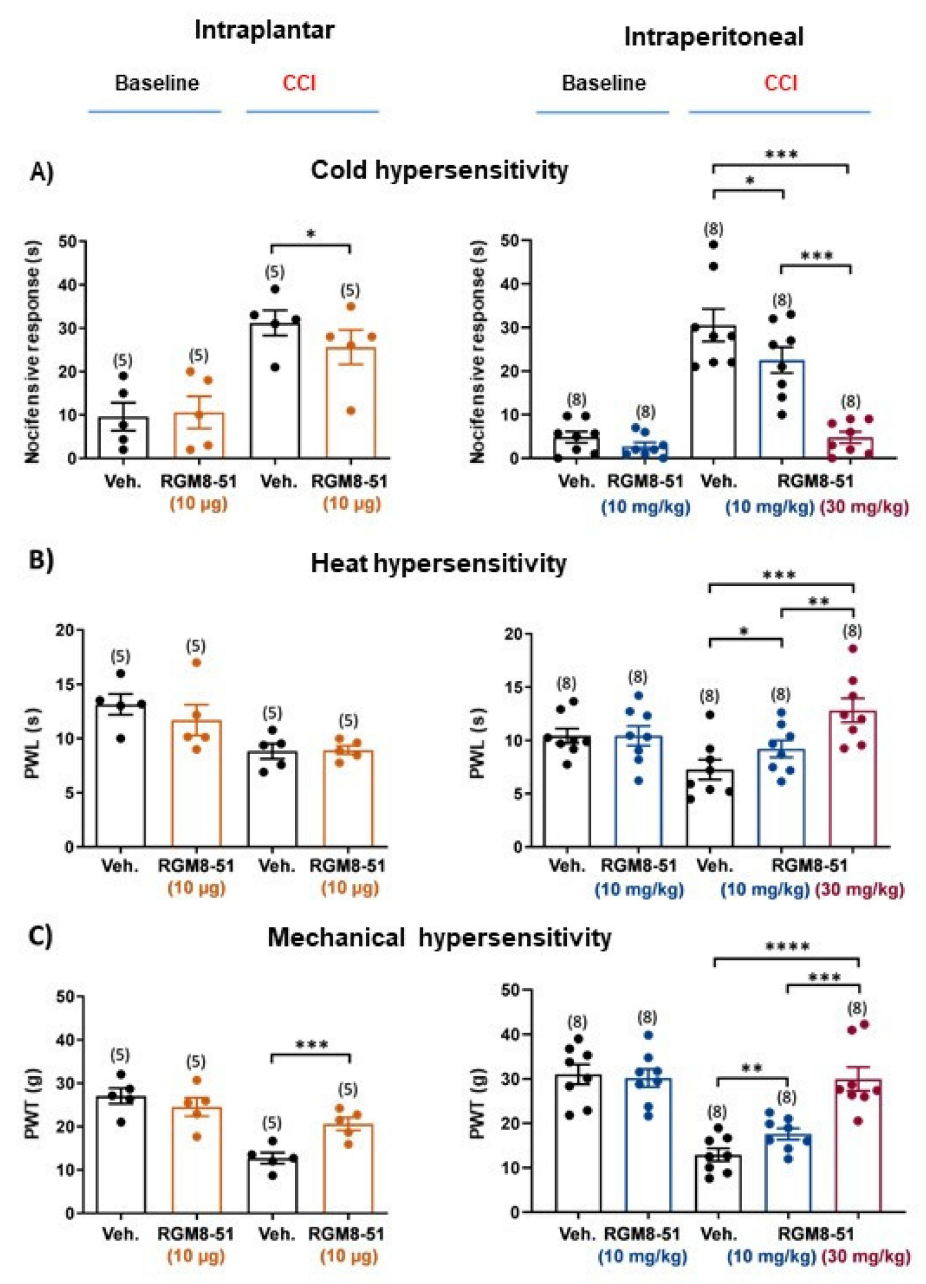
Effects of compound RGM8-51 on cold, heat and mechanical hypersensitivity in the CCI model in the rat. RGM8-51 was administered before (baseline) and after CCI surgery (CCI) by both the intraplantar (10 µg, left panels) and the intraperitoneal (10 and 30 mg/kg, right panels) routes. Note that vehicle administration after CCI surgery was associated to an increase in the nocifensive response duration to acetone application, and to reductions in the paw withdrawal latency (PWL) to heat radiation and the paw withdrawal threshold (PWT) to tactile stimulation of the injured hind paw, indicative of cold (**A**), heat (**B**) and mechanical (**C**) hypersensitivity, respectively. Data are mean ± SEM of the number of animals specified between parentheses. Statistical analysis was performed by using the paired *t*-test. * < 0.05; ** < 0.01; *** < 0.001; **** < 0.0001 with regard to vehicle (Veh.) or between the two i.p. doses.

The CCI model of neuropathic pain allowed the evaluation of the effect RGM8-51 on cold, heat and mechanical sensitivity [5], as assayed by the acetone test, the Hargreaves test, and the automatized von Frey test, respectively. RGM8-51 was applied either locally (i.pl.; 10 µg) in the affected paw or systemically (intraperitoneally, i.p., 10 and 30 mg/kg) and the effect evaluated before (Baseline) and after CCI surgery. The nocifensive response after administration of RGM8-51 was compared with that of vehicle, while non-operated animals and the uninjured (contralateral) hind paw in CCI animals were used as controls. As expected from previous results on the role of TRPM channels and the effect of TRPM8 antagonists (DFL23448, DFL23693, IGM-18) in cold hypersensitivity in CCI animals [12,13], RGM8-51 reduced cold hypersensitivity following i.pl. application, and inhibited it dose-dependently after i.p. administration (Figure 7A). Interestingly, no effect was observed in either non-operated animals (baseline in Figure 7A) or the contralateral uninjured paw (data not shown).

Compound RGM8-51 was also effective in decreasing hypersensitivity to heat following i.p. administration and it did it dose-dependently (Figure 7B). This result is in line with those obtained with β-carboline-based TRPM antagonists in CCI mice by using the thermal ring assay, which measures thermal preference behavior [11]. Curiously, i.pl. application of RGM8-51 did not influence the animal response to heat, which points towards the possibility that the effect of RGM8-51 could be related to body temperature regulation rather than heat sensing. In fact, TRPM8 antagonists are known to decrease the body temperature, a phenomenon that is abrogated in TRPM^-/-^ mice [9,13,51,52]. As it was the case for cold stimulation, RGM8-51 had no effect on heat perception in non-operated animals (baseline in Figure 7B) or in the uninjured hind paw (data not shown).

Last, we tested the effect of RGM8-51 on mechanical sensitivity. Again, this compound dose-dependently reverted paw withdrawal thresholds after i.p. administration, this effect also being produced after local (i.pl.) delivery, whereas it could not be observed in the absence of neuropathy (Figure 7C).

Altogether, the results obtained with RGM8-51 in the CCI model point to a multi-sensory modality (cold, heat, and tactile) antinociceptive effect, without affecting normal sensory perception. Likewise, the fact that the effects on cold and mechanical sensitivity were observed after i.pl. application suggests the involvement of a peripheral site of action, most likely the skin terminals of the small sized (C-type) DRG neurons, which are specialized in nociceptive transduction. This possibly provides an explanation for the seemingly contrasting results obtained when TRPM8 receptors are downregulated or blocked by intrathecal administration of TRPM8 antisense oligonucleotides or antagonists (AMTB), where no effect could be observed on mechanical allodynia in CCI rats [6,9].

Notably, a writhing behavior was observed in a small number of animals following i.p. injection of 30 mg/kg RGM8-51. This behavior lasted 3-5 min and could possibly be related to the TRPV1 agonist activity of compound RGM8-51.

#### 2.4.3. NTG-Induced Mechanical Hypersensitivity

Recently, it has been shown that both CGRP receptors and different TRP ion channels (TRPM8, among them) are associated to the development of migraines [53]. In addition, treatment with TRPM8 agonists has been claimed to alleviate topically the symptoms of this pathology [54], whereas the clinical development of the oral antagonist, AMG333, was stopped due to serious side effects, mainly related to body temperature dysregulation [29]. To the best of our knowledge, apart from AMG333, no other TRPM8 antagonists have been evaluated in vivo for migraine. Since β–lactam RGM8-51 possesses analgesic activity in other animal models, we considered also its evaluation in a mouse model of chronic migraine characterized by mechanical hypersensitivity and induced by administration of nitroglycerin (NTG), a known vasodilator used in the treatment of angina pectoris [55]. This model has been used to evaluate the effects of known acute and preventive migraine therapies and to demonstrate the role of miR155-5p upregulation in the pathological mechanism of chronic migraine [56,57].

Administration of NTG intermittently produces a baseline hypersensitivity to mechanical stimulation, which can be measured by the von Frey test. The mechanical stimulus was applied at different times to obtain the time course of the effects produced after compound administration, and both male and female mice were used to investigate a possible sex dimorphism.

**Figure 8:**
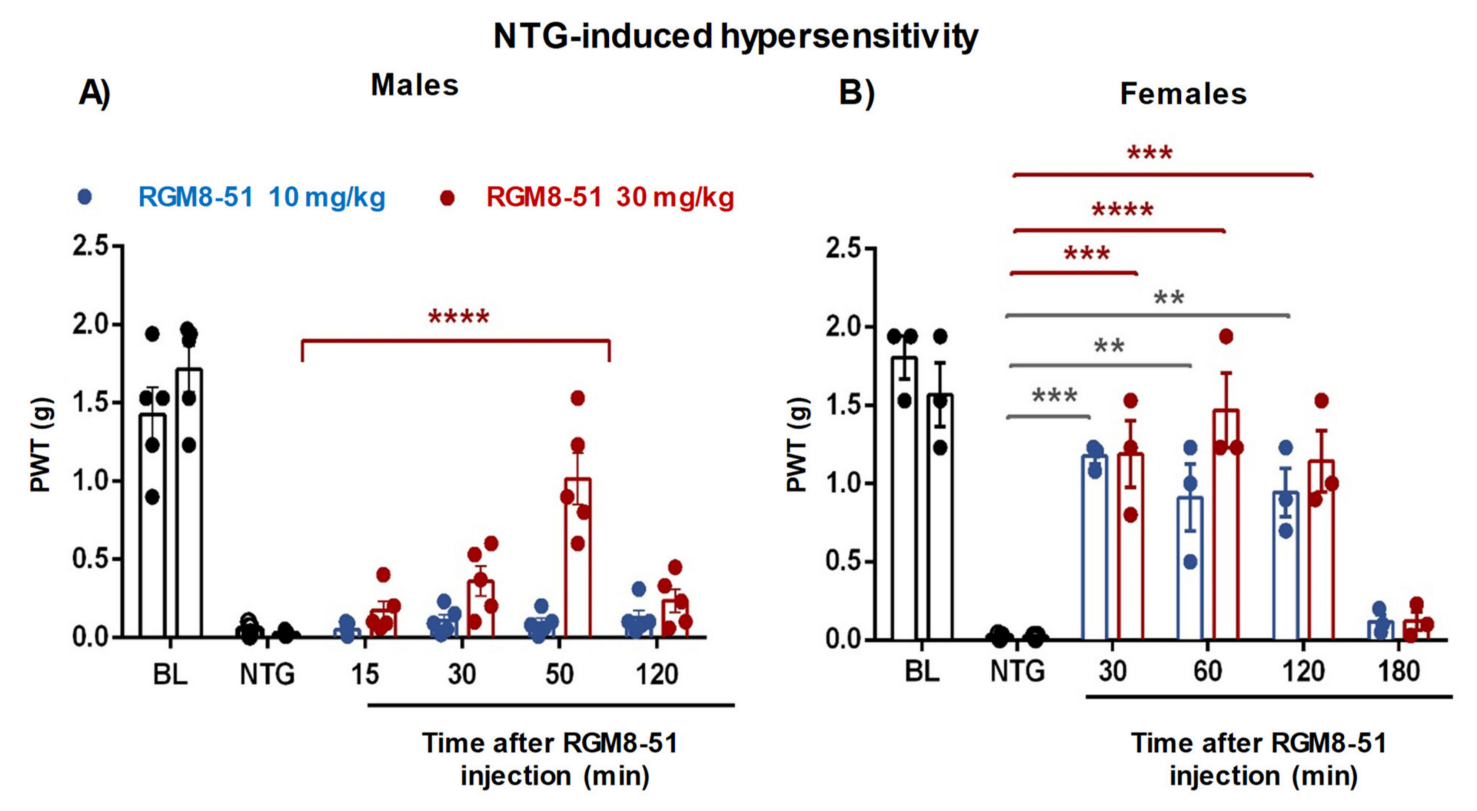
β–lactam RGM8-51 reduces NTG-induced hypersensitivity in a sex-dependent manner. (**A**) Paw withdrawal threshold (PWT) increased dose-dependently in C57 male mice 2 h after injection of 30 mg/kg of NTG. (**B**) NTG-induced mechanical hypersensitivity was fully blocked in female mice by the systemic injection of RGM8-51 compound in a dose dependent manner. Compound RGM8-51 was intraperitoneally injected 2h after NTG injection and 30 min before von Frey measurements. Data are given ± SEM (n = 3–5). Statistical analysis was two-way ANOVA followed by post hoc Bonferroni test by multiple comparison ** *p* < 0.05; *** *p* < 0.001; **** *p* < 0.0001.

Figure 8A graphically compiles the results obtained in male mice. Thus, it can be observed that the administration of NTG decreases the mechanical stimulation that the animals are capable of bearing, compared to controls without NTG. This NTG-induced hypersensitivity can only be significantly blocked by RGM8-51 at a 30 mg/kg i.p. dose, 60 min post-administration. Figure 8B shows the results of the same test carried out in female mice. In this case, compound RGM8-51 significantly reduces the hypersensitivity threshold induced by NTG at both 10 and 30 mg/kg i.p. doses. In addition, the observed positive effect takes place faster, with significant antinociceptive activity at 30 min, and is of longer duration (even after 120 min post-administration). This differential behavior could be explained by the fact that TRPM8 acts as a fast integrator of the testosterone hormone, an agonist of the channel [58,59]. As testosterone is mainly found in males, the activation of the TRPM8 receptor by this hormone could counteract the effect of the β–lactam. Actually, previous results with TRPM8^-/-^ male and female mice demonstrated that TRPM8 is involved in the regulation of dimorphic sexual and social behaviors [60].

## 3. Conclusions

TRPM8 channels in sensory neurons have been involved in different pain processes. However, the dissimilar results found with different TRPM8 antagonists demand new studies to establish the future dimension of TRPM8 channel inhibition for pain relief. To shed light on the participation of TRPM8 channels in different animal models of pain, we selected a prototype from our family of β–lactams, RGM8-51. In in vitro experiments, we demonstrated that this compound has good TRPM8 antagonist activity (rat and human isoforms), and appreciable selectivity versus other thermoTRP channels and pain-mediating receptors. Additionally, in DRG neurons, RGM8-51 inhibited menthol-activated currents, while it did not have a direct effect on major ion conductances involved in action potential generation. In vivo experiments evidenced that compound RGM8-51 was highly effective in different TRPM8-mediated types of pain. The inhibition of cold hypersensitivity in oxaliplatin-induced peripheral neuropathy anticipates the potential utility of this type of compound in the disabling condition associated to chemotherapeutic cancer treatments. Additionally, in the CCI model of neuropathic pain, this β–lactam was shown to attenuate cold and mechanical hypersensitivity and had some effect on heat stimulation. Lastly, RGM8-51 mitigates, in a sex-dependent manner, the NTG-induced mechanical hypersensitivity. Despite non-optimal pharmacokinetic properties, RGM8-51 was revealed as a helpful chemical tool for studying TRPM8-mediated pharmacology after i.pl. and i.p. administration. These results also ratify the 1,3,4,4-tetrasubstituted β–lactam ring as a valuable central scaffold in the search for new TRPM8 modulators. Further research on this family of TRPM8 antagonists is ongoing in our labs.

## 4. Methods

### 4.1. Synthesis

*General procedures*: Reactions were monitored either by TLC and/or analytic HPLC (UV, detection, 220 nm). Flash columns, filled with silica gel Merck 60 (230–400) were used for chromatographic separations. A reversed-phase column, Sunfire C18 (4.6 × 50 mm, 3.5 µm), a flux of 1 mL/min, and mixtures of CH_3_CN, phase A, and H_2_O, phase B, both containing 0.01% formic acid, were used for analytical HPLCs. Mass spectra, in electrospray, positive mode, were obtained on a Waters Micromass ZQ spectrometer. High resolution mass spectrum (ESI-HRMS) was recorded on an Agilent 6520 Q-TOF instrument. Optical rotation for final compound RGM8-51 was measured in a polarimeter Perkin Elmer 141 apparatus. NMR spectra were recorded in a Varian INOVA-400 (400 MHz) spectrometer, operating at 400 and 100 MHz for ^1^H and ^13^C experiments, respectively (with chemical shifts expressed in ppm and coupling constants in Hz).

#### 4.1.1. Ns-L-Phe-O*^t^*Bu (**5**)

To a solution of H-L-Phe- O*^t^*Bu.HCl (11.64 mmol, 3.00 g) in dry DMF (70 mL), triethylamine (TEA) (11.64 mmol, 1.61 mL) was added, and the reaction stirred 20 min at rt. Then, the reaction mixture was cooled to 0 °C, a second portion of TEA (12.22 mmol, 1.69 mL) and o-nosylchloride (12.22 mmol, 2.71 g) were added, and stirred overnight at rt. The solvent was evaporated, and the reaction mixture was dissolved in EtOAc, and successively washed with citric acid (10%), NaHCO_3_ (10%) and brine. The organic layer was dried over Na_2_SO_4_, filtered and evaporated to dryness. The final product was precipitated in cold Et_2_O, and the precipitate filtered. Product still remaining in mother liquids was purified on a silica gel column, using 11% EtOAc in hexane as eluent. A total of 4.37 g (93%) of compound **5** was obtained as a white solid. Mp: 90 °C (AcOEt:hexano). Published: 92–93 °C [61]. HPLC: t_R_ = 8.73 min (gradient from 30% to 95% of A, in 10 min). ^1^H-NMR (300 MHz, CDCl_3_): *δ* 8.02 (m, 1H, Ar), 7.87 (m, 1H, Ar), 7.68 (m, 2H, Ar), 7.30 –7.13 (m, 5H, Ar), 6.02 (d, 1H, *J* = 9.1 Hz, αNH), 4.35 (m, 1H, α-Phe), 3.09 (m, 2H, β-Phe), 1.19 (s, 9H, *^t^*Bu). MS (ES)^+^: 351.10 [M-*^t^*Bu]^+^.

#### 4.1.2. N-[(2*S*-Dibenzylamino-3-phenyl)prop-1-yl]-Ns-L-Phe-O^t^Bu (**7**)

To a solution of 2*S*-dibenzylamino-3-phenyl-1-propanol (**6**) (7.49 mmol, 2.48 g), Ns-L-Phe-O*^t^*Bu (**5**) (8.24 mmol, 3.35 g) and triphenylphosfine (8.24 mmol, 2.16 g) in dry THF (60 mL), under Ar atmosphere at 0 °C, diisopropyl azodicarboxyate (8.24 mmol, 1.62 mL) was added. Then, the reaction was stirred at rt overnight. The solvent was removed under vacuum, and the resulting residue was purified on a silica gel column, using a gradient from 66 to 80% DCM in hexane. Compound **7** (4.94 g, 92%) was obtained as a syrup. HPLC: t_R_ = 5.00 min (gradient from 80% to 95% of A, in 10 min). ^1^H-NMR (300 MHz, CDCl_3_): *δ* 7.59 (m, 2H, Ar), 7.37 (m, 1H, Ar), 7.27-7.16 (m, 18H, Ar), 7.08 (m, 2H, Ar), 6.86 (m, 1H, Ar), 4.51 (t, 1H, *J* = 7.7 Hz, α-Phe), 4.01 (dd, 1H, *J* = 15.0, 5.6 Hz, H_1_), 3.81 (d, 2H, *J* = 13.7 Hz, NCH_2_), 3.63 (d, 2H, *J* = 13.8 Hz, NCH_2_), 3.45 (dd, 1H, *J* = 15.0, 8.8 Hz, H_1_), 3.29 (ddd, 1H, *J* = 8.6, 5.8, 2.8 Hz, H_2_), 2.83 (m, 2H, H_3_), 2.75 (m, 2H, β-Phe), 1.1 1 (s, 9H, CH_3_, *^t^*Bu). ^13^C-NMR (75 MHz, CDCl_3_): *δ* 169.0 (COO), 148.2, 140.1, 139.9, 136.8, 134.6, 133.3, 132.0, 129.7, 129.5, 128.4, 121.3, 128.2, 127.0, 126.9, 126.1, 124.4 (Ar), 82.0 (C, *^t^*Bu), 62.6 (α-Phe), 57.9 (C_2_), 53.3 (NCH_2_), 47.0 (C_1_), 37.1 (β-Phe), 34.7 (C_3_), 27.7 (CH_3_, *^t^*Bu). MS (ES)^+^: 721.38 [M + H]^+^.

#### 4.1.3. N-[(2*S*-Dibenzylamino-3-phenyl)prop-1-yl]-L-Phe-O*^t^*Bu (**8**)

To a solution of the *N*-nosyl-*N*-alkyl derivative **7** (6.77 mmol, 4.87 g) in dry ACN (125 mL) K_2_CO_3_ (20.30 mmol, 2.80 g) and phenylthiol (13.53 mmol, 1.38 mL) were added slowly and stirred at rt overnight. After evaporation of the solvent in vacuum, the resulting residue was dissolved in EtOAc:H_2_O (1:1) and the phases were separated. The organic layer was dried over Na_2_SO_4_, filtered and evaporated to dryness. The resulting mixture was purified on a silica gel column, using a gradient from 9 to 33% of EtOAc in hexane. Compound **8** (3.56 g 98%) was obtained as a syrup. HPLC: t_R_ = 4.50 min (gradient from 15% to 95% of A in 5 min). ^1^H-NMR (300 MHz, CDCl_3_): *δ* 7.37–7.17 (m, 18H, Ar), 7.02 (m, 2H, Ar), 3.77 (d, 2H, *J* = 13.7 Hz, NCH_2_), 3.59 (d, 2H, *J* = 13.7 Hz, NCH_2_), 3.32 (dd, 1H, *J* = 7.9, 5.8 Hz, α-Phe), 3.02 (m, 2H, H_2_, H_3_), 2.92 (dd, 1H, *J* = 13.6, 5.9 Hz, β-Phe), 2.82 (dd, 1H, *J* = 13.4, 7.8 Hz, β-Phe), 2.70-2.45 (m, 3H, H_1_, H_3_), 1.78 (NH),1. 33 (s, 9H, CH_3_, *^t^*Bu). ^13^C-NMR (75 MHz, CDCl_3_): *δ* 173.5 (COO), 140.4, 140.0, 138.0, 129.6, 129.4, 128.9, 128.5, 128.4, 128.4, 126.9, 126.7, 125.9 (Ar), 81.0 (C, *‘*Bu), 64.0 (α-Phe), 60.3 (C_2_), 53.9 (NCH_2_), 47.5 (C_1_), 39.4 (β-Phe), 34.1 (C_3_), 28.1 (CH_3_, *^t^*Bu). MS (ES)^+^: 536.16 [M + H]^+^.

#### 4.1.4. N-[(2*S*-Dibenzylamino-3-phenyl)prop-1-yl]-N-(2′*S*-chloropropanoyl)-L-Phe-O*^t^*Bu (**9**)

A solution of 2(*S*)-chloropropionic acid (11.47 mmol, 1.067 mL) and trichloroacetonitrile (12.62 mmol, 1.265 mL), in anhydrous THF (35 mL), was cooled to 0 °C and triphenylphosphine (12.61 mmol, 3.31 g) was slowly added. Then, the reaction mixture was stirred at rt for 1h. This solution was slowly added, at 0 °C, to a second solution containing the secondary amine derivative **8** (5.73 mmol, 3.06 g) and propylene oxide (114.69 mmol, 2.666 mL) in anhydrous THF (10 mL). The evolution of the reaction was controlled by analytical HPLC. Once completed, the solvent was evaporated, the resulting residue was dissolved in EtOAc, and washed with citric acid (10%), NaHCO_3_ (10%) and brine. The organic layer was dried over Na_2_SO_4_, filtered and evaporated in vacuum. Purification was performed on silica gel column, using as eluent a gradient from 6 to 9% of EtOAc in hexane. Compound **9** (3.40 g, 95%) was obtained as a syrup. HPLC: t_R_ = 5.61 min (gradient from 60% to 95% of A in 10 min). ^1^H-NMR (400 MHz, DMSO-d^6^, rotamers ratio M:m, 12:1): *δ* major rotamer 7.30–7.10 (m, 12H, Ar), 6.90 (m, 8H, Ar), 4.09 (q, 1H, *J* = 6.5 Hz, H_1′_), 3.64 (dd, 1H, *J* = 9.5, 5.6 Hz, α-Phe), 3.56 (d, 2H, *J* =13.8 Hz, NCH_2_), 3.45 (d, 2H, *J* =13.8 Hz, NCH_2_), 3.25 (dd, 1H, *J* = 14.2, 4.8 Hz, H_1_), 3.19 (dd, 1H, *J* = 14.2, 9.6 Hz, H_1_), 3.04 (m, 3H, β-Phe, H_2_), 2.82 (dd, 1H, *J* = 13.6, 4.9 Hz, H_3_), 2.65 (dd, 1H, *J* = 13.6, 9.3 Hz, H_3_), 1.54 (d, 3H, *J* = 6.5 Hz, H_2′_), 1. 44 (s, 9H, CH_3_, *^t^*Bu). ^13^C-NMR (75 MHz, DMSO-d^6^): *δ* major rotamer 169.9 (COO), 168.8 (CON), 139.3, 139.2, 139.1, 138.6, 129.8, 129.4, 128.8, 128.7, 128.3, 127.3, 126.7, 126.4 (Ar), 82.0 (C, *^t^*Bu), 64.9 (α-Phe), 58.3 (C_2_), 53.7 (NCH_2_), 52.3 (C_1_), 49.9 (C_1′_), 35.5 (β-Phe), 34.8 (C_3_), 28.1 (CH_3_, *^t^*Bu), 21. 3 (C_2′_). MS (ES)^+^: 625.43 [M + H]^+^.

#### 4.1.5. 4(*S*)-Benzyl-3*S*-methyl-4-(*tert*-butoxy)carbonyl-1-[(2′*S*-dibenzylamino-3′-phenyl)prop-1′-yl]-2-oxoazetidine (RGM8-51, **3**)

A solution of compound **9** (5.15 mmol, 3.22 g) in dry ACN (20 mL), was treated, under Ar atmosphere, with BTPP (7.73 mmol, 2.360 mL) and stirred at rt until the chloropropanoyl derivative disappeared. Then, the solvent was evaporated and the residue dissolved in EtOAc and washed with 0.1 M HCl and brine. The organic layer was dried over Na_2_SO_4_, filtered and evaporated in vacuum. Purification was performed on silica gel column, using as eluent a gradient from 9 to 50% EtOAc in hexane. Compound 3 (2.43 g, 80%) was obtained as a syrup. HPLC: t_R_ = 2.33 min (gradient from 50 to 95% of A in 5 min). [α]_D_ = −1.64 (c 1, CH_3_Cl). ^1^H-NMR (400 MHz, CDCl_3_): *δ* 7.31–7.04 (m, 20H, Ar), 3.76 (d, 2H, *J* = 13.9 Hz, NCH_2_), 3.56 (d, 2H, *J* = 13.8 Hz, NCH_2_), 3.57 (m, 2H, H_1′_, H_2′_), 3.42 (m, 1H, H_1′_), 3.14 (q, 1H, *J* = 7.5 Hz, H_3_), 2.95 (m, 3H, H_3′_, 4-CH_2_), 2.92 (d, 1H, *J* = 14.3 Hz, 4-CH_2_), 1.46 (s, 9H, CH_3_, *^t^*Bu), 1.19 (d, 3H, *J* = 7.6 Hz, 3-CH_3_). ^13^C-NMR (75 MHz, CDCl_3_): *δ* 170.2 (COO), 169.7 (C_2_), 140.2, 139.6, 135.6, 129.8, 129.5, 128.6, 128.3, 128.1, 127.9, 127.0, 126.8, 125.7 (Ar), 82.8 (C *^t^*Bu), 68.3 (C_4_), 58.0 (C_2′_), 53.3 (NCH_2_), 53.1 (C_3_), 42.7 (C_1′_), 41. 3 (4-CH_2_), 36.4 (C_3′_), 28.0 (CH_3,_ *^t^*Bu), 10.7 (3-CH_3_). MS (ES)^+^: 589.47 [M + H]^+^. Exact mass calculated for C_39_H_44_N_2_O_3_: 588.33519, found 588.33556. Before biological assays, compound **9** was transformed into its hydrochloride salt by treatment with 0.1. M HCl (1 equiv) in a mixture of ACH/H_2_O (1:1), followed by lyophilization. ^1^H-NMR (400 MHz, Methanol-*d*_4_) δ 7.79 (m, 1H, Ar), 7.60 (m, 3H, Ar), 7.43 (m, 3H, Ar), 7.33 (m, 3H, Ar), 7.24 (m, 7H, Ar, NH), 7.12 (m, 2H, Ar), 6.99 (m, 2H, Ar), 5.10 (d, *J* = 12.9 Hz, 1H, NCH_2_), 4.53 (d, *J* = 13.6 Hz, 1H, 4-CH_2_), 4.45 (d, *J* = 13.6 Hz, 1H, 4-CH_2_), 4.30 (d, *J* = 12.9 Hz, 1H, NCH_2_), 4.16 (m, 1H, H_1′_), 4.02 (dd, *J* = 16.6, 10.6 Hz, 1H, H_1′_), 3.56 (m, 1H, H_2′_), 3.49 (d, *J* = 14.6 Hz, 1H, NCH_2_), 3.22 (q, *J* = 7.6 Hz, 1H, H_3_), 2.96 (d, *J* = 14.3 Hz, 1H, NCH_2_), 2.72 (t, *J* = 12.9 Hz, 1H, H_3′_), 2.51 (d, *J* = 15.8 Hz, 1H, H_3′_), 1.23 (s, 9H, CH_3_, *^t^*Bu), 0.88 (d, *J* = 7.6 Hz, 3H, 3-CH_3_).

### 4.2. Molecular Modeling Studies

Molecular modeling studies started from the TRPM8 cryo-EM structure of *Ficedula albicollis* (PDB code: 6BPQ [45]), as previously described [30]. The homology modeling of rat TRPM8 was performed on completed TRPM8 sequence (Uniprot code Q8R455), using the *Ficedula albicollis* structure as template, and adding the missing loops with the standard protocol implemented in the program Yasara [43,44,59] (version 20.12.24). The sequence alignment between *Ficedula a.* and rat TRPM8 was performed with ClustalO [60], a service located in the European Bioinformatics Institute EMBL-EBI (https://www.ebi.ac.uk/ accessed on 2 December 2020). Docking studies were accomplished with AutoDock [61] implemented in Yasara and consisted in a total of 800 flexible global docking runs that were set and clustered around the putative binding sites, following by a simulated annealing optimization of each generated complex, using the force field AMBER03 (Assisted Model Building with Energy Refinement) [62]. The best binding energy complex in each cluster was analyzed, stored, and selected as the best orientation of the interacting partners. Edition, modeling and visualization of the molecules were performed with Yasara (http://www.yasara.org accessed on 2 December 2020). Figures were drawn with Pymol, the open source version of the PyMOL Molecular Graphics System, version 2.4.1 (Schrödinger, LLC, New York, NY, USA) obtained at http://www.pymol.org accessed on 2 December 2020.

### 4.3. Functional Assays by Calcium Microfluorimetry

To measure the effect of the compounds against TRPM8 activity we used microfluorometry-based calcium flux assays with Fluo-4 NW Ca^2+^ dye and fluorescence as described in [30]. Briefly, human embryonic kidney cell line (HEK293) cells stably transfected with rTRPM8 or hTRPM8 were seeded in 96-well plates at a cell density of 30,000 cells. After 2 days the medium was replaced with 100 μL of the dye, Fluo-4 NW, loading solution supplemented with probenecid 2.5 mM and incubated 1 h at 37 °C. The TRPM8 activity was measured using POLASTAR plate reader (BMG Labtech) setting the excitation wavelength at 485 nm and the emission wavelength at 520 nm. The baseline fluorescence was recorded for 3 cycles before the addition of vehicle compound at different concentrations and the antagonist, 10 μM AMTB. Fluorescence intensity was recorded during 7 more cycles and 100 μM menthol was added. Fluorescence intensity was recorded during 10 more cycles.

Data analysis: The Z-factor was calculated in each assay using the following equation: (3 × (SDmax + SDmin))/(Mean max-Mean min). In all the experiments, the Z-factor was ≥0.5. The effect of the compounds against TRPM8 activity was determined by normalizing their effect on the maximum fluorescence observed after application of 100 μM menthol. Decrease in menthol signal was expressed as percentage of inhibition (%). All data are expressed as the mean ± standard deviation (SD). Data are expressed as the concentration exerting a half-maximal inhibition of agonist-induced [Ca^2+^]i elevation (IC_50_), which was calculated again using GraphPad Prism® software. All determinations were performed in triplicate (n = 3) in 3 independent experiments (N = 3).

### 4.4. Isolation of Rat DRG Neurons

Voltage-activated Na^+^ and K^+^ currents and TRPM8-mediated currents were recorded in DRG neurons isolated from 6-8 weeks old Sprague-Dawley rats, as previously described [62]. Rats were sacrificed by cervical dislocation followed by decapitation and lumbar segments of the spinal column were removed and placed in a cold Ca^2+^, Mg^2+^-free Hank’s solution (Sigma-Aldrich, Madrid, Spain). The bone surrounding the spinal cord was removed and the right L4, L5 and L6 DRG were exposed and pulled out. After removing the roots, DRG were chopped in half and incubated for 30 min at 37°C in Dulbecco’s modified Eagle’s Medium-low glucose (DMEM; Sigma-Aldrich) containing 5 mg/mL collagenase XI (Sigma-Aldrich, Madrid, Spain), 100 U/mL penicillin (Sigma-Aldrich), and 0.1 mg/mL streptomycin (Sigma-Aldrich). Then, the cell suspension was washed with DMEM by centrifugation (300 G, 5 min at 4 °C), filtered through a 100 µm mesh to eliminate cell clumps and washed again by centrifugation. The cell pellet was resuspended in DMEM and 40 µL were dropped onto 10 mm diameter glass coverslips treated with poly-L-lysine (1 mg/mL, 30 min; Sigma-Aldrich) placed in 35 mm diameter Petri dishes. Finally, plated cells were flooded with 2.5 mL of DMEM supplemented with 10% fetal calf serum (Sigma-Aldrich, Madrid, Spain), 100 U/mL penicillin and 0.1 mg/mL streptomycin, and stored in an incubator (Hera Cell, Heraeus, Hanau, Germany) with a 5% CO_2_/95% air atmosphere at 37 °C. This protocol yields spherical cell bodies without neurites, from which only small to medium DRG neurons (diameter < 30 μm) were chosen for electrophysiological recording within 12–24 h of plating.

Changes in membrane potential were studied in neonatal DRGs isolated from 3–5 days-old Wistar rats. After isolation, DRGs were digested first using collagenase type IA (0.25% *w*/*v*) in DMEM GlutaMax with penicillin/streptomycin (P/S) solution (1% *v*/*v*) for 1 h (37 °C, 5% CO_2_, ThermoScientific incubator) and secondly by mechanical dissociation. Single cell suspension was passed through a 100 μM cell strainer and washed with DMEM GlutaMax with FBS (10%*v*/*v*) and P/S solution (1% *v*/*v*). Cells were seeded in a drop on 24-well plates containing 12 mm Ø glass coverslips pretreated with poly-L-lysine (8.3 μg/mL) and laminin (5 μg/mL). After 1 h, medium was replaced with DMEM GlutaMax with FBS (10%*v*/*v*) and P/S solution (1% *v*/*v*), supplemented with mouse 2.5S NGF (50 ng/mL) and cytosine arabinoside (1.25 μg/mL). All cell culture procedures were performed in a laminar flow cabinet (Model Telstar AV-100, Institut Català de Nanociència i Nanotecnologia, Bellaterra, Spain). Experiments were performed 24-48 h after cell seeding [63].

### 4.5. Functional Assays by Patch-Clamp Electrophysiology

Whole-cell patch-clamp recordings from HEK293-rTRPM8 cells were carried out 2 days after seeding on 12 mm Ø glass coverslips treated with poly-L-lysine solution (Sigma Aldrich, Spain) [30]; the intracellular pipette solution contained (in mM) 150 NaCl, 5 EGTA, 3 MgCl_2_ and 10 HEPES, adjusted to pH 7.2 with NaOH, and the extracellular solution contained (in mM) 150 NaCl, 6 CsCl, 1.5 CaCl_2_, 1 MgCl_2_, 10 D-glucose and 10 HEPES, adjusted to pH 7.4 with NaOH. The TRPM8 activity was measured by the application of two pulses of 100 μM menthol in a time interval of 2 min and a 30 s perfusion of the different compound concentrations before the second menthol pulse.

The extracellular solution used in whole-cell voltage-clamp recordings from rat DRG neurons contained (mM) 145 NaCl, 2.8 KCl, 3 CaCl_2_, 1 MgCl_2_, 10 4-(2-Hydroxyethyl)piperazine-1-ethanesulfonic acid, N-(2-Hydroxyethyl) piperazine-N′-(2-ethanesulfonic acid) (HEPES), and 12 glucose (pH 7.35 adjusted with NaOH; ≈320 mOsm); the internal solution contained (mM) 145 KCl, 2 MgCl_2_, 0.3 EGTA, 0.3 GTP.Li_3_, 2 ATP.Na_2_, 10 HEPES (pH 7.2 adjusted with KOH; ≈310 mOsm). Menthol (100 µM) or RGM8-51 (1 µM; 2-4 min superfusion) were applied directly onto the cell under investigation by means of a multibarrel concentration-clamp device coupled to electronically driven miniature solenoid valves under the control of PatchMaster software (HEKA Electronics, Lambrecht, Germany). Voltage-activated currents were elicited by a step depolarization to +10 mV, 100 ms long, associated to a P/4 protocol for on-line leak and capacitive current subtraction.

For current-clamp recordings from rat DRG neurons the intracellular pipette solution contained (in mM): 4 NaCl, 126 K gluconate, 0.02 CaCl_2_, 1 MgSO_4_, 5 HEPES, 15 glucose, 3 ATP, 0.1 GTP and 5 EGTA, pH 7.2 with KOH and the extracellular solution contained (in mM): 140 NaCl, 4 KCl, 2 CaCl_2_, 2 MgCl_2_, 10 HEPES, 5 glucose and 20 mannitol, pH 7.4 with NaOH. The membrane potential was measured in the absence and the presence of compound RGM8-51 at 10 μM in a time interval of 1 min.

Data were sampled at 10 kHz (EPC10 amplifier with PatchMaster 2.53 software, HEKA Electronics, Lambrecht, Germany) and low-pass filtered at 3 kHz for analysis (PatchMaster 2.53 and GraphPad Prism 6.0, Graphpad Software, San Diego, CA, USA). The series resistance was <10 MΩ and to minimize voltage errors was compensated by 60–80%. All measurements were performed at 24–25 °C. Cell capacitance was measured and used to estimate the current density (J, pA/pF).

Data analysis: For the whole-cell patch-clamp experiments, results are expressed as the percentage of remaining activation of the TRPM8 channel. This is calculated by normalizing the ratio (p2/p1) of testing conditions to the ratio (p2/p1) of the control condition. Analysis of the data was performed by GraphPad 6.0, the Ordinary One-Way ANOVA analysis followed by the post hoc Bonferroni test for multiple comparisons and the ROUT method (Q = 10%) identified data outliers. A non-linear regression curve and IC_50_ were obtained by the representation of log (inhibitor) vs. response. Paired or unpaired Student’s *t*-tests were also used for data comparison between two groups. All data are expressed as the mean ± standard error of the mean (SEM) (n = 5–8). In the current-clamp experiments, the change in membrane potential is expressed as the difference between the resting membrane potential and the membrane potential after the addition of a stimuli. Data were analyzed as previously described (GraphPad 6.0, one-way ANOVA followed by Bonferroni multiple comparisons test, and ROUT method, Q = 10%).

### 4.6. Selectivity and Preliminary PK Properties

These studies were subcontracted to Eurofins-CEREP or Eurofins-PANLABS. Briefly, agonists and antagonist assays were performed in cell cultures overexpressing the corresponding channel (CHO: TRPV1 and TRPA1; HEK293: TRPV3 and ASIC3). In vitro pharmacology, cellular channel functional assays: the compound was prepared in assay buffer to the final concentration, 10 μM. The compound wells, reference agonist and background vehicle controls were prepared in DMSO at 0.3%. The reference agonist for each Ion Channel assayed was prepared in a similar manner to serve as the assay control. The reference agonist for each Ion Channel was included at Emax (the concentration where the reference agonist elicited a maximal response). The agonist assay was conducted on fluorimeter instruments where the test compound, vehicle controls, and reference agonist were added to the assay plate after a fluorescence baseline was established. The agonist assay was used to assess each compound’s ability to activate each Ion Channel assayed. For the antagonist assay, the compound was pre-incubated for five minutes at room temperature, and then challenged with the EC80 concentration of the reference agonist after establishment of a fluorescence baseline. Vehicle controls and EC80 concentration of reference agonist were added to appropriate wells. The antagonist assay was used to assess each compound’s ability to inhibit activation by the corresponding Ion Channel agonist. ASIC3 ionflux antagonist assay: all recordings were obtained from a holding potential of -60 mV. The compound addition sequence was as follows: one addition of pH5.5 buffer was added to establish the baseline response, the test concentration of compound was applied for 30 s, followed by the addition of pH5.5 of compound for 2 s. Binding assays were performed in CHO cell cultures overexpressing human CGRPR, CB2 and M2 receptors. RGM8-51 (10 μM) was tested, and reference compounds used as control agonists and antagonists are detailed in Table 2, footnote. Binding assay results are expressed as a percent of control specific binding. Preliminary PK analysis, namely solubility, log D, in vitro Caco-2 permeability, human binding to plasma protein and in vitro metabolism (human liver microsomes) was performed at Eurofins-CEREP, using established methods. Aqueous solubility (μM) was determined by comparing the peak area of the principal peak in a calibration standard (200 μM) containing organic solvent (methanol/water, 60/40, *v*/*v*) with the peak area of the corresponding peak in a buffer sample. Partition coefficient: the total amount of compound was determined as the peak area of the principal peak in a calibration standard (100 μM) containing organic solvent (methanol/water, 60/40, *v*/*v*). The amount of compound in buffer was determined as the combined, volume-corrected, and weighted areas of the corresponding peaks in the aqueous phases of three organic-aqueous samples of different composition. An automated weighting system was used to ensure the preferred use of raw data from those samples with well quantifiable peak signals. The amount of compound in organic phase was calculated by subtraction. Subsequently, Log D was calculated as the Log10 of the amount of compound in the organic phase divided by the amount of compound in the aqueous phase. Protein binding: percentage of compound bound to serum proteins. Permeability was measured in CACO-2 monolayers. Percentage of compounds in the receptors wells was measured using HPLC. Lucifer yellow was used to assess cell monolayer integrity. In vitro metabolism was measured in human liver microsomes. The half-life (t_1/2_) was estimated from the slope of the initial linear range of the logarithmic curve of compound remaining (%) vs. time, assuming the first-order kinetics. The apparent intrinsic clearance (CLint, in μL/min/mg) was calculated from the half-life time.

### 4.7. Mouse Model of Oxaliplatin-Induced Peripheral Neuropathy

For the experiments, 6–8 weeks C57 male mice (≈30g) (Harlam, Holland) were used. All experiments were approved by the Institutional Animal and Ethical Committee of the Universidad Miguel Hernandez and Comunidad Valencian ethical committees (2021/VSC/PEA/0089) where experiments were conducted and they were in accordance with the guidelines of the Economic European Community and the Committee for Research and Ethical Issues of the International Association for the Study of Pain. All parts of the study concerning animal care were performed under the control of veterinarians. Experiments were performed blinded for NTG and compound treatment.

Oxaliplatin (OXA, Tocris, Bristol, UK) was used to induce peripheral neuropathy and was dissolved in water with gentle warming as formerly described [34]. OXA was intraperitoneally injected on days 1, 3 and 5 at a 6 mg/kg dose. On day 7, experiments were carried out. Additionally, and in order to prevent kidney damage or dehydration, animals were also i.p. injected with saline solution and a 5% mannitol solution. The compound RGM8-51 stock was prepared in DMSO (Sigma-Aldrich) and freshly diluted in saline just before injections. RGM8-51 compound was injected i.pl. at different doses (0.1–1 μg) in a volume of 25 μL in the right hind paw of mice. Cold chemical sensitivity was assessed using the acetone drop test. Briefly, animals were kept in a metal mesh cage and allowed to habituate for approximately 30 min in order to acclimatize them. An acetone drop (10 μL) was applied on the plantar surface of one of the hind paws. Positive nociceptive responses (licking time in seconds) were recorded with a digital stopwatch during 20 s (cut-off). Acetone was applied twice in an interval of 5 min between each application and the mean was calculated.

### 4.8. Rat Chronic Constriction Injury (CCI) Model

Adult male Sprague-Dawley rats (weighing 200–220 g/6–8 weeks old) were used in these experiments. All experimental procedures were conducted according to the animal welfare guidelines of the European Community (European Directive 2010/63/UE) to minimize animal suffering and were approved by Universidad Complutense de Madrid and Comunidad de Madrid ethical committees on animal experimentation (PROEX 207.8/21).

CCI was performed according to Bennett and Xie (1988) [5]. Briefly, rats were anesthetized with i.p. ketamine (100 mg/kg; Merial Labs, Barcelona, Spain) and medetomidine (100 µg/kg; Esteve Labs, Barcelona, Spain). Under sterile conditions, approximately 7 mm of the right nerve was freed proximal to the sciatic trifurcation, and four barely constricting ligatures (1 mm apart) using 4/0 chromic catgut were applied. The incision was closed in layers with silk thread 6/0. Animals were then allowed to recover from surgery for 7 days before being used in additional procedures.

For behavioral testing, rats were placed in individual methacrylate boxes with a wire mesh floor, and after a period of 15 min acclimatization, the cold, heat and mechanical sensitivity of the ipsilateral and contralateral paws was determined. Cold sensitivity was assessed with the acetone test. In total, 100 µL acetone were locally applied to the plantar surface of the hind paw and the duration of the subsequent nocifensive response (licking, brushing, and flinching of the paw) was determined during 60 s. Acetone application and assessment were repeated 2 times with a 5 min interval and the sum of the two values was recorded. Heat sensitivity was determined with the Hargreaves test, which uses infrared light as a heat source, with a Hargreaves apparatus (Serie 8, Model 336, IITC Life Science, Woodland Hills, CA USA). The light beam was directed to the plantar surface of the hind paw and the time from the beginning of irradiation to the appearance of first paw withdrawal movement was measured (paw withdrawal latency). A cut-off of 20 s was set to prevent tissue injury. Two measurements were taken separated by a 1 min interval, and the mean value was calculated. Mechanical sensitivity was evaluated with a dynamic plantar aesthesiometer (Ugo Basile, Gemonio, Italy) by means of a 0.5 mm filament exerting increasing force (up to 50 g over 20 s) onto the plantar surface of the hind paw until the animal lifted its paw, the actual force at that time was automatically registered (paw withdrawal threshold). Measurements were repeated 3 times at 5 min intervals, and the mean value was reported. Behavioral tests were carried out before surgery (mean of 3 measurements on alternate days the week preceding surgery, collectively designated as baseline) and on days 7–21 post-surgery.

Rats were allocated into 2 groups to receive RGM8-51 and vehicle (DMSO 2% i.pl., or 150 µL/kg i.p.) by either the i.pl. (10 µg in 20 µL; n = 5 animals) or i.p. (10 or 30 mg/kg in 150 µL/kg; n = 8 animals) route. Responses to cold/heat/mechanical stimulation were assessed 30 min before and 30 min after RGM8-51 or vehicle injection. Behavioral testing prior to drug administration served to ascertain baseline conditions and the stability of cold, heat and mechanical hypersensitivity after surgery. RGM-8-51 stock was prepared in DMSO (Sigma-Aldrich) and diluted in saline for injections. Data are given as the mean ± standard error of the mean (SEM) of the corresponding number of behavioral measurements/animals used. Statistical analysis was performed by using the paired *t*-test (GraphPad 8; San Diego, CA, USA).

### 4.9. Mouse Model of NTG-Induced Mechanical Hypersensitivity

C57BL6 mice (males and females, ≈25 g) (Envigo, Blackthorn, UK) were used for the study. All experiments were approved by the Institutional Animal and Ethical Committee of the Universidad Miguel Hernandez where experiments were conducted and they were in accordance with the guidelines of the Economic European Community and the Committee for Research and Ethical Issues of the International Association for the Study of Pain. All parts of the study concerning animal care were performed under the control of veterinarians. Commercially available nitroglycerin (NTG) formulation (Nitro Pohl, 10 mg/kg) was administered i.p. 2h before measurements. The NTG stock solution was diluted in saline up to a final dose of 10 mg/kg, which was injected to the animals. RGM-8-51 stock was prepared in DMSO (Sigma-Aldrich) and diluted in saline for injections. Compound was i.p. administered. The first von Frey measurement was performed 30 min after RGM8-51 injection, and subsequently at the indicated times (60, 120, and 180 in females). Mechanical threshold values were obtained by performing the von Frey test as previously reported [1]. Mice were placed on a wire mesh platform and, after a 20 min habituation period, von Frey filaments (Stoelting, Wood Dale, IL, USA) were applied to the plantar side of the paws. von Frey filaments 2.44, 2.83, 3.22, 3.61, 4.08 and 4.56 were used and, starting with the 3.61 filament, 6 measurements were taken in each animal randomly starting by the left or right paw. Based on the “up and down” method [2], the observation of a positive response (lifting, shaking or licking of the paw) was followed by the application of the immediate thinner filament or the immediate thicker one if the response was negative. The 50% response threshold was calculated using the following formula:

50% (g) threshold = (10^(*Xf* + *kd*)^)/10,000

where *Xf* is the value of the last von Frey filament applied; *k* is a correction factor based on pattern of responses (from the Dixon’s calibration table); *d* is the mean distance in log units between stimuli (here, 0.4).

Von Frey threshold for each animal was calculated, according to the formula, and data analysis was performed by using GraphPad 6.0. Ordinary two-way ANOVA analysis followed by the post hoc *Bonferroni* test for multiple comparisons.

## Figures and Tables

**Table 1:** Antagonist activity of RGM8-51 at *r*TRPM8 and *h*TRPM8 channels (Ca^2+^ microfluorography and patch-clamp assays).

Compd.	Ca^2+^ Microfluorography Assays				Patch-Clamp Assay
	*r*TRPM 8 IC_50_ (μM)	Intervals	*h*TRPM8 IC_50_ (μM)	Intervals	*r*TRPM8 IC_50_ (μM)
RGM8-51 (**3**)	1.06 ± 1.21	0.72 to 1.55	1.74 ± 1.19	1.23 ± 2.45	0.97 ± 1.56
**2**	3.1 ± 1.1	2.57 to 3.99	_	_	0.9 ± 1.0
AMTB	7.3 ± 1.5 *		6.23 ± 0.02 **		_

*r*TRPM8: transient receptor potential melastatin, type 8 channel, rat isoform. *h*TRPM8: transient receptor potential melastatin, type 8 channel, human isoform. Three different experiments, performed in triplicate. * Reference [31]. ** *Versus* icilin (Reference [33]).

**Table 2:** Functional activity of RGM8-51 (10 μM) in Ca^2+^ microfluorography assays of TRPV1, TRPV3, TRPA1 and ASIC3 channels, and percentage of inhibition of specific binding at CGRPR, CB2 and M3 receptors.

Compd.	*h*TRPV1 ago. (%)	*h*TRPV1 antago. (%)	*h*TRPV3 antago. (%)	*h*TRPA1 ago. (%)	*h*TRPA1 Antago. (%)	ASIC3 Antago. (%)	Binding *h*CGRPR (%)	Binding *h*CB_2_ (%)	Binding *h*M_3_ (%)
RGM8-51	31.3 ± 3.6	19.4 ± 1.7	−6.3 ± 5.9	0.4 ± 0.3	4.3 ± 1.1	6.0 ± 0.9	−1.0 ± 4.3	7.3 ± 0.3	−4.1 ± 3.1

*h*TRPV1: transient receptor potential vanilloid, type 1. *h*TRPV3: transient receptor potential vanilloid, type 3. *h*TRPA1: transient receptor potential ankirin, type 1. ASIC3: acid sensing ion channel, subunit 3. *h*CGRPR: human calcitonin gen-related peptide receptor. *h*CB_2_: human cannabinoid receptor, subtype 2. hM3: human muscarin receptor, subtype 3. In all cases, data is from two experiments in duplicate. Ago.: assay for agonist activity. Antago.: assay for antagonist activity (see methods). Agonists used for activation: TRPV1 (Capsaicin, 30 nM), TRPV3 (2-Aminoethoxydiphenyl borate, 2-APB, 30 µM), TRPA1 (Allylisothiocyanate, 10 µM), ASIC3 (Buffer, pH 5,5). Reference antagonists: TRPV1 (Capsazepin, CI_50_ 1.3∙10^−7^), TRPV3 (Ruthenium red, CI_50_ 2.5∙10^−7^ M), TRPA1 (Ruthenium red, 10 µM), ASIC3 (Amiloride, 1 mM). Radioligand for hCGRPR: [^125^]hGCRPα, agonist *h*GCRP_α_ (1 µM). Radioligand for hCB_2_: [^3^H]WIN 55212-2, agonist WIN 55212-2 (5 µM). Radioligand for hM_3_: [^3^H]4-DMAP, agonist 4-DMAP (1 µM).

**Table 3:** Main sites found for compound RGM8-51 and statistical distribution of docking solutions.

Subsite	Location	% of Docking Solutions (Estimated Binding Energies, kcal/mol)
1	Inner pore, S5S6, S5	58% (8.39)
2	Internal mouth pore	12% (8.44)
3	External pore, S3S4, S6	16% (6.63)
4	External loops	8% (6.65)

## Supplementary Materials

The following supporting information can be downloaded at: https://www.mdpi.com/article/10.3390/ijms23052692/s1.
