# Supplementary material for: β–Lactam TRPM8 Antagonist RGM8-51 Displays Antinociceptive Activity in Different Animal Models
Source: Int J Mol Sci. 2022 Feb 28;23(5):2692. doi: 10.3390/ijms23052692 (PMC8910920; doi:10.3390/ijms23052692)
Supplement: Supplementary file 1 [file ijms-23-02692-s001.zip › ijms-1591571-supplementary.pdf]

β-Lactam TRPM8 antagonist RGM8-51 displays antinociceptive activity in different animal models

Cristina Martín-Escura, Alicia Medina-Peris, Luke A. Spear and Roberto de la Torre Martínez, Luis A. Olivos-Oré, María Victoria Barahona, Sara González-Rodríguez, Gregorio Fernández-Ballester, Asia Fernández-Carvajal, Antonio R. Artalejo, Antonio Ferrer-Montiel, Rosario González-Muñiz

Supporting Information

Table S1. Preliminary pharmacokinetic profile of **RGM8-51**.

Compound	Solubility (μM)[a] in simulated gastric fluid	Log D	Permeability A-B[b] (%)	Permeability B-A[b] (%)	Binding to proteins (%)	Metabolism $t_{1/2}$ (min)[c]	Metabolism Clint[c] (μL/min/mg)
**RGM8-51**	198.3	5.77	0	0	100	11.3	613.6
**Warfarin**					99		
**Tamoxifen**		4.64					
**Terfenadine**						7.0	971.9
**Propanolol**			70	74		>60	<115

Studies performed at Eurofins-Cerep following their standard assays (see experimental methods for details). One experiment in duplicate

[a] Aqueous solubility (μM). Values: 193.99, 202.64.

[b] CACO-2 cell monolayer. A-B, percentage of compound in the acceptor cell. Values 0.01-0.04

[c] Liver microsomes culture. Values: 11.6, 11.0.
